# Asymmetric atomic-level engineering of phosphorus-modified Cu single-atom coordination centers on carbon nitride for enhanced photocatalytic hydrogen evolution

**DOI:** 10.1039/d6sc04795e

**Published:** 2026-07-30

**Authors:** Hafijul Islam, Bhavya Jaksani, Saad Mehmood, Sukanya Saha, Bidyut Bikash Sarma, B. Moses Abraham, Ujjwal Pal

**Affiliations:** a Department of Energy & Environmental Engineering, CSIR-Indian Institute of Chemical Technology Hyderabad Telangana 500007 India upal03@gmail.com ujjwalpal@iict.res.in; b Academy of Scientific and Innovative Research (AcSIR) Ghaziabad 201002 India; c University of Toulouse, Toulouse INP, CNRS, LCC Toulouse France; d A. J. Drexel Nanomaterials Institute, Department of Materials Science and Engineering, Drexel University Philadelphia 19104 USA; e Department of Chemical Engineering, Indian Institute of Technology Kanpur Kanpur-208016 India

## Abstract

Single-atom photocatalysts (SACs) have emerged as an effective strategy for enhancing solar energy conversion by improving light absorption and charge carrier dynamics; however, precise construction of isolated active sites remains challenging. Herein, we report atomically dispersed Cu single atoms uniformly anchored on phosphorus-doped graphitic carbon nitride (Cu-PCN). The optimized catalyst achieves an excellent hydrogen evolution rate of 3276 µmol g^−1^ h^−1^ with an apparent quantum yield (AQY) of 31% at 400 nm, surpassing most reported metal–N coordinated systems. X-ray absorption spectroscopy (XAS) confirms the atomic dispersion and defined coordination of Cu single atoms within the g-C_3_N_4_ framework. Combined experimental and theoretical studies reveal that the Cu sites extend light absorption, enhance charge separation and interfacial transfer, and promote H_2_O activation by guiding electron migration toward Cu centers with reduced energy barriers, thereby facilitating the formation of H* intermediates as the crucial step in hydrogen evolution. Overall, this work demonstrates an effective approach to engineer asymmetric active sites *via* coordination tuning, providing valuable insights for designing efficient photocatalysts for solar-driven hydrogen evolution reaction.

## Abstract 2

Asymmetric atomic-level engineering and synergistic interactions between adjacent sites significantly enhance photocatalytic hydrogen evolution by improving light absorption and charge-carrier dynamics.

## Graphical abstract

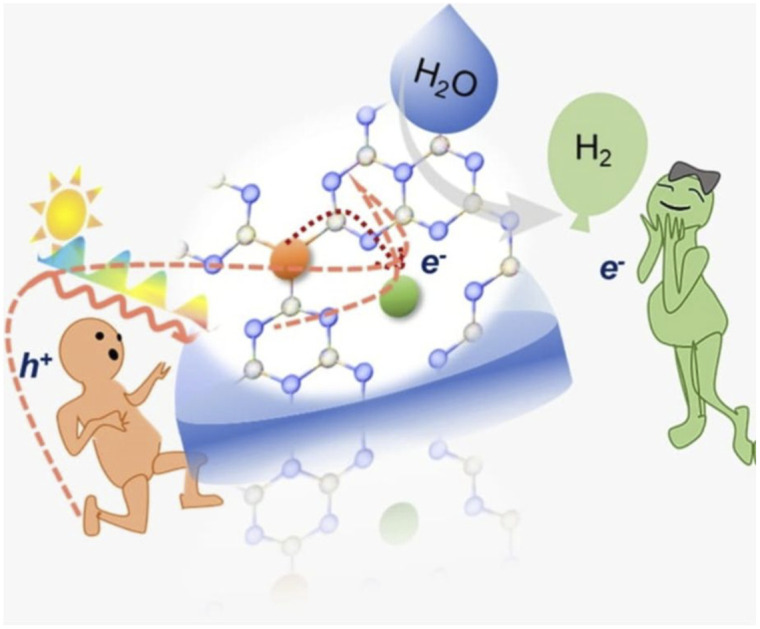

## Introduction

The extensive utilization of fossil fuels has led to severe environmental pollution and an intensifying global energy crisis, thereby necessitating the development of clean and sustainable energy alternatives. Hydrogen, possessing an exceptionally high gravimetric energy density of 142 MJ kg^−1^, is widely recognized as a promising renewable energy carrier.^1–4^ Among the various hydrogen production technologies, solar-driven photocatalytic water splitting has attracted considerable attention as an efficient and sustainable approach for clean energy generation. Since the pioneering discovery of the photocatalytic effect in 1972, semiconductor-based photocatalysts have been widely investigated, resulting in significant advancements in this field.^5–8^ Within this context, graphitic carbon nitride (g-C_3_N_4_) has emerged as a highly attractive photocatalyst owing to its visible-light responsiveness, excellent chemical stability, environmental benignity, low cost, and strong potential for large-scale applications. However, pristine g-C_3_N_4_ is hindered by rapid recombination of photogenerated charge carriers, resulting in inherently low photocatalytic efficiency and significantly limiting its practical and industrial utilization.^9–12^

Various modification strategies-such as heteroatom doping, heterojunction construction, morphological engineering, and defect modulation have been extensively explored to enhance charge separation and transfer in g-C_3_N_4_-based photocatalysts.^13,14^ However, these strategies typically do not result in significant improve exciton dissociation and long-range charge transport, as the short-range lattice structure of g-C_3_N_4_ tends to localize charge carriers.^15^ To address this limitation, atomically dispersed metal sites have been incorporated into g-C_3_N_4_, where isolated single atoms such as Pt, Ru, Fe, Co, Ni, and Cu anchored on the framework act as efficient electron acceptors and thereby facilitate rapid charge transfer.^16–18^ Their atomic dispersion ensures nearly complete metal atom utilization while promoting in-plane charge migration, leading to enhanced photocatalytic efficiency.^19–21^ Similarly, heteroatom doping (*e.g.*, S, P, and B) elegantly tunes the band gap of g-C_3_N_4_, enhances visible-light absorption, and tailors its coordination environment, thereby inducing asymmetric charge distribution and enabling more directed electron transfer.^22,23^

Photocatalytic performance can be enhanced through first-sphere modification (direct coordination changes) and second-sphere engineering (heteroatom introduction near active sites); notably, second-sphere modification generates asymmetric redox centers that create local electric fields, promote charge redistribution, suppress recombination, and improve both the selectivity and activity of g-C_3_N_4_-based photocatalysts.^24–26^ Zhou *et al.* prepared Cu single-atom-anchored porous g-C_3_N_4_*via* a one-step co-heating method. The optimized catalyst exhibited an H_2_ evolution rate of 2142.4 µmol h^−1^ g^−1^ under visible light (*λ* > 420 nm), which was 15 and 109 times higher than that of porous and bulk g-C_3_N_4_, respectively, owing to the formation of Cu–N coordination bonds.^27^ Li *et al.* reported louver-like P-doped g-C_3_N_4_ nanowires with an HER rate of 1872.9 µmol h^−1^ g^−1^ and an AQY of 6.93% at 420 nm, representing a 25.6-fold enhancement over bulk g-C_3_N_4_.^28^ He *et al.* reported an asymmetric Cu–B dual-atom configuration on two-dimensional g-C_3_N_4_, where the synergistic interaction between adjacent Cu and B atoms optimized the electronic structure and significantly promoted the photocatalytic activity.^29^ Li *et al.* developed a B–Pt–O asymmetric coordination structure on TiO_2_, where the synergistic interaction between Pt single atoms and Pt nanoparticles optimized electron distribution and significantly enhanced photocatalytic hydrogen evolution.^30^

Inspired by recent advances in single-atom catalysis and asymmetric coordination engineering, this work employs a facile phosphidation strategy to introduce P atoms, enabling the formation of asymmetrically coordinated single-atom Cu sites in g-C_3_N_4_ (Cu-PCN). Unlike previously reported Cu single-atom anchored g-C_3_N_4_ systems or asymmetric coordination structures on oxide supports, the present study integrates phosphorus-induced electronic modulation with Cu single-atom anchoring on the g-C_3_N_4_ framework, thereby simultaneously regulating the local coordination environment, defect chemistry, and interfacial charge-transfer pathways. The optimized Cu-PCN photocatalyst exhibits a remarkable H_2_ evolution rate of 3276 µmol g^−1^ h^−1^ under visible-light irradiation without any noble-metal cocatalyst or photosensitizer, along with an apparent quantum yield of 31% at 400 nm and excellent long-term stability. Combined experimental characterization and density functional theory calculations reveal that the synergistic interaction between P dopants and isolated Cu atoms broadens visible-light absorption, promotes charge separation and migration, and optimizes hydrogen adsorption with a near-ideal Δ*G*_H*_, thereby significantly accelerating the hydrogen evolution reaction. This work establishes an effective asymmetric atomic-level engineering strategy for designing highly efficient and stable g-C_3_N_4_-based photocatalysts for solar hydrogen production.

## Results and discussion

### Synthesis and structural characterization

**Fig. 1:**
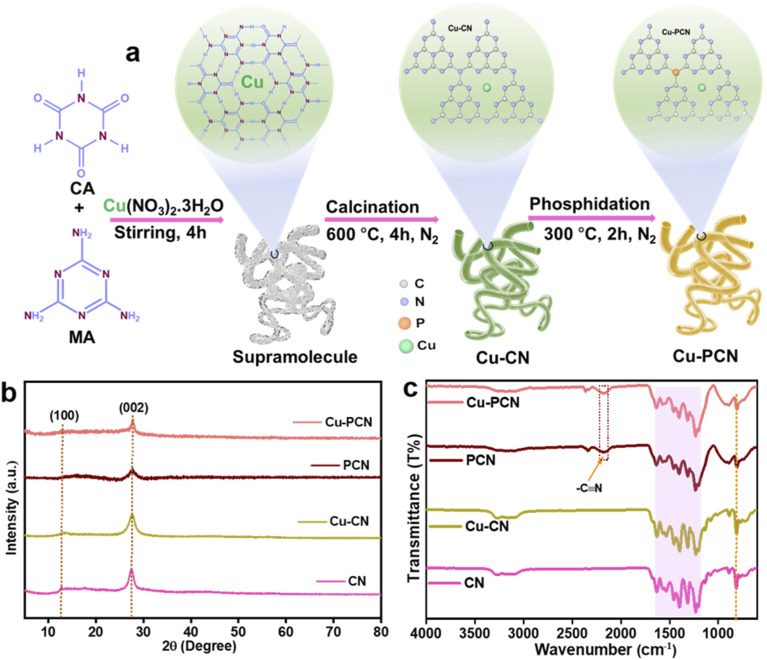
(a) Schematic representation of the synthesis route for Cu-CN and Cu-PCN. (b) PXRD patterns and (c) FTIR spectra of CN, Cu-CN, PCN, and Cu-PCN samples.

Atomically dispersed Cu single atoms on P-doped g-C_3_N_4_ (Cu-PCN) were synthesized *via* a two-step thermal polymerization strategy, as illustrated in Fig. 1a. Initially, melamine, cyanuric acid, and Cu (NO_3_)_2_·3H_2_O were dissolved in water and self-assembled into a coordination-linked supramolecular precursor, ensuring uniform dispersion of Cu species. Upon drying, the precursor was thermally polymerized to form Cu single-atom anchored g-C_3_N_4_ (Cu-CN). Subsequently, a phosphidation step introduced P atoms into the second sphere environment, ultimately yielding the desired asymmetric Cu-PCN catalyst. The detailed synthesis procedure is provided in the SI. The PXRD patterns of the as-prepared photocatalysts (Fig. 1b) show two characteristic peaks at ∼13.0° and ∼27.5°, assigned to the (100) and (002) planes of CN, corresponding to the in-plane tri-*s*-triazine units and interlayer stacking structure, respectively. Notably, Cu-PCN, Cu-CN, and PCN retain similar diffraction features to pristine CN, indicating that the framework remains largely preserved after Cu incorporation and thermal treatment. However, the reduced peak intensity observed for PCN and Cu-PCN suggests a partial disruption of structural order induced by the phosphidation process.^24^ The FTIR spectra (Fig. 1c) show a characteristic peak at ∼810 cm^−1^, assigned to the out-of-plane bending vibration of heptazine (C–N=C) units. The bands in the 1200–1700 cm^−1^ region correspond to C–N heterocyclic stretching, while the broad absorption at 3000–3400 cm^−1^ is attributed to N–H stretching vibrations.^31^ Notably, Cu-CN, PCN, and Cu-PCN show similar absorption features to pristine CN, confirming that the fundamental framework remains intact after Cu incorporation and phosphidation. The Cu contents in Cu-CN and Cu-PCN were determined by ICP-MS to be 0.49 and 0.47 wt% respectively, as summarized in Table S1. Furthermore, CHNS elemental analysis (Table S2) reveals that the C/N ratio of PCN (0.59) is slightly lower than that of pristine CN (0.61). This subtle decrease suggests the generation of carbon vacancies in PCN, likely induced by phosphorus incorporation.

**Fig. 2:**
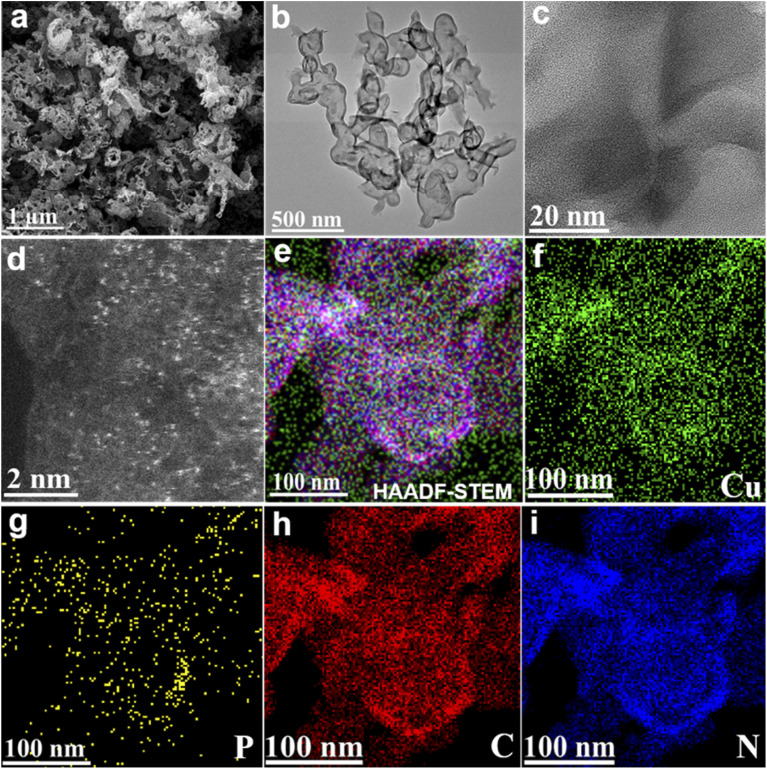
(a) SEM image, (b) TEM image, (c) HRTEM image, and (d) HAADF-STEM image of Cu-PCN. (e–i) Corresponding EDX elemental mapping images showing the distribution of Cu, P, C, and N, respectively.

The morphology and structural features of the as-synthesized Cu-PCN samples were examined using scanning electron microscopy (SEM) and transmission electron microscopy (TEM). As evidenced by the SEM images (Fig. 2a and S1a) and TEM images (Fig. 2b and S1b), the samples exhibit a distinct twisted tubular morphology. Notably, the incorporation of Cu single atoms along with P does not cause any appreciable change in the morphology of the CN framework (Fig. S2). High-resolution TEM (Fig. 2c) further verifies the absence of metallic or metal oxide nanoparticles, suggesting that Cu species are atomically dispersed within the Cu-PCN catalyst. Aberration-corrected high-angle annular dark-field scanning transmission electron microscopy (AC-HAADF-STEM) images display numerous isolated bright spots uniformly distributed over the nanotube support, which can be ascribed to atomically dispersed Cu sites in Cu-PCN (Fig. 2d) and Cu-CN (Fig. S3). In addition, EDS elemental mapping of Cu-PCN (Fig. 2e–i) confirms the homogeneous distribution of Cu, P, C, and N throughout the nanotubular framework.

**Fig. 3:**
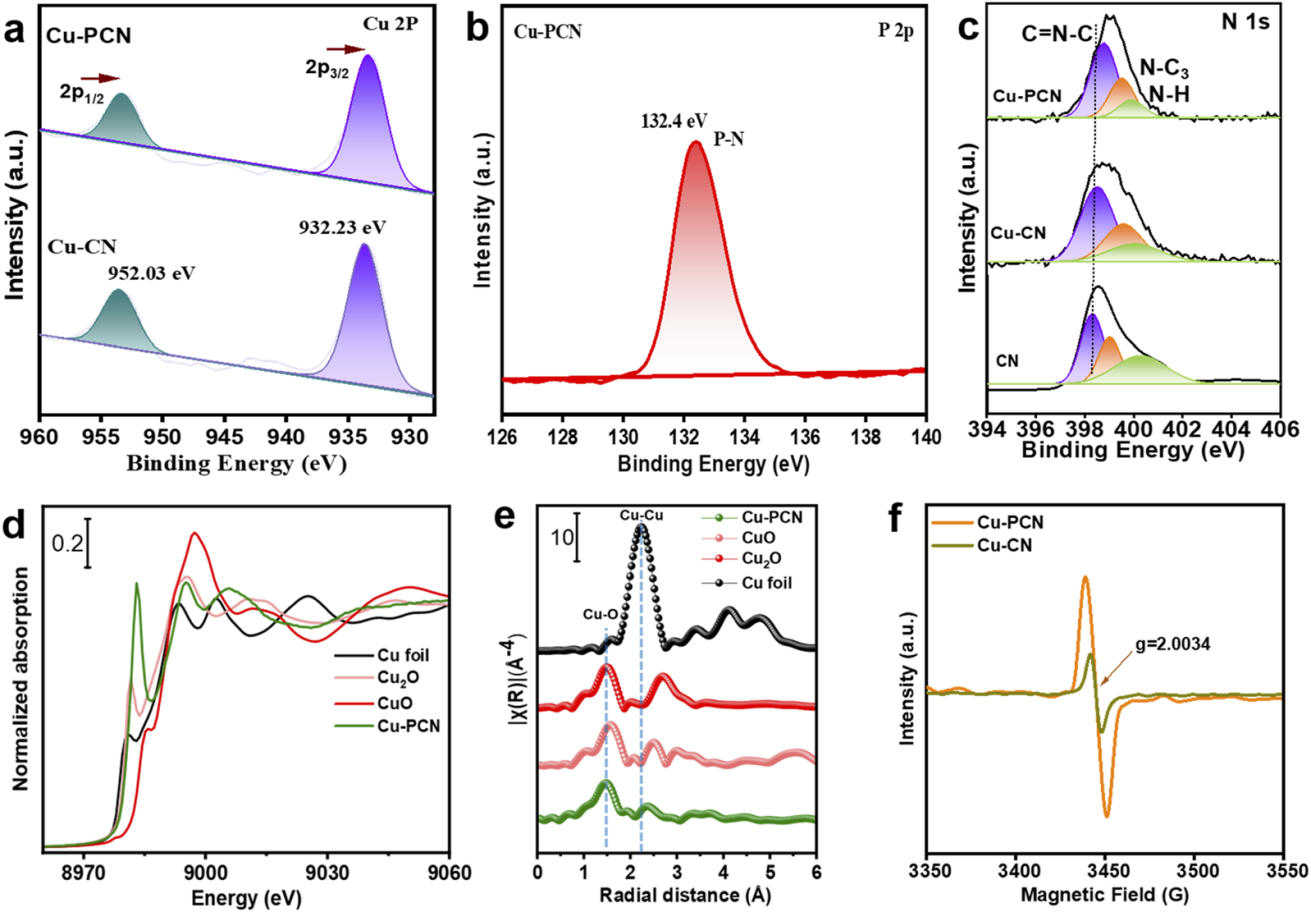
(a) High-resolution XPS spectra of Cu 2p for Cu-CN and Cu-PCN; (b) high-resolution XPS spectra of P 2p for Cu-PCN; (c) high-resolution XPS spectra of N 1s for CN, Cu-CN, and Cu-PCN; (d) Cu K-edge XANES spectra of Cu foil, Cu_2_O, CuO, and Cu-PCN; (e) *k*^3^-weighted Fourier transform EXAFS spectra at the Cu K-edge for the corresponding samples; and (f) EPR spectra of Cu-CN and Cu-PCN.

The surface elemental composition and chemical states of the synthesized samples were systematically investigated by X-ray photoelectron spectroscopy (XPS). The high-resolution Cu 2p spectrum (Fig. 3a) shows two prominent peaks located at 932.23 and 952.03 eV, corresponding to Cu 2p_3/2_ and Cu 2p_1/2_, respectively. The absence of characteristic shake-up satellite peaks suggests that Cu predominantly exists in Cu^0^ or Cu^+^ oxidation states.^32^ In addition, the P 2p spectrum (Fig. 3b) presents a distinct peak at 132.4 eV, which can be attributed to P–N bonding, thereby confirming the successful incorporation of phosphorus into the heptazine framework.^28^ The absence of a P–C bonding peak, which typically appears 1–2 eV lower than that of the P–N signal, indicates that P atoms are incorporated into the g-C_3_N_4_ framework by substituting C atoms within the heptazine units.^33^ Compared to Cu-CN, the Cu 2p peak in Cu-PCN exhibits a shift toward lower binding energy, indicating a strong electronic interaction between P and Cu species in the Cu-PCN framework. The high-resolution C 1s spectrum of the samples (Fig. S4) displays three characteristic peaks at 284.8 eV, 286.2 eV and 288.6 eV, which are assigned to C–C, C–N and sp^2^-hybridized N–C=N species, respectively.^34,35^ The N 1s spectrum (Fig. 3c) can be deconvoluted into three distinct components centered at 398.4, 399.7, and 400.8 eV, corresponding to pyridinic nitrogen (C=N–C), tertiary nitrogen N–(C)_3_ units, and amino-type nitrogen (N–H/NH_*x*_), respectively.^36^ The N–H peak area decreases from 23.24% for pristine CN to 16.34% for Cu-PCN, as summarized in Table S3. This reduction indicates the formation of nitrogen vacancies in Cu-PCN.^37,38^ Notably, in both Cu-CN and Cu-PCN, the pyridinic N peak serving as the primary coordination site for Cu exhibits a shift toward higher binding energy compared to pristine CN, indicating electron transfer from nitrogen atoms to the Cu centers.

X-ray absorption spectroscopy (XAS) was utilized to confirm the atomic nature of the Cu-PCN catalyst. The Cu K-edge X-ray absorption near-edge structure (XANES) spectra are shown in Fig. 3d; the absorption edge position of Cu-PCN is located between those of Cu foil and CuO, and is closer to Cu_2_O, indicating that the Cu species predominantly exist in a positively charged Cu^1+^ state.^39^ The presence of a strong pre-edge peak of Cu-PCN due to the symmetry forbidden 1s to 3d transition indicates that the Cu is in a highly non-centrosymmetric environment. A strong feature at 9005 eV indicates the presence of a heavier scatterer which can be due to the phosphorus. The Fourier transformed extended X-ray absorption fine structure (FT-EXAFS) analysis showed that there is a predominant Cu–N shell present at a distance of 1.5 Å (Fig. 3e). Importantly, no characteristic Cu–Cu scattering peak around 2.2 Å is detected, confirming the absence of Cu clusters or metallic nanoparticles and demonstrating the atomic dispersion of Cu sites. In addition, the *k*-space EXAFS oscillation patterns of Cu-PCN (Fig. S5) are distinctly different from those of Cu foil, Cu_2_O, and CuO, further indicating the formation of unique Cu coordination environments in the Cu-PCN catalyst. These findings collectively verify that isolated Cu atoms are successfully anchored within the phosphorus-modified g-C_3_N_4_ framework, forming asymmetrically coordinated Cu active centers. The electron paramagnetic resonance (EPR) spectra display a characteristic signal at *g* = 2.0034 for Cu-CN (Fig. 3f), confirming the presence of nitrogen-related defects. Notably, Cu-PCN exhibits a significantly stronger EPR signal, indicating a higher concentration of defect sites. This enhanced defect density is attributed to phosphorus incorporation and the elevated calcination temperature, both of which promote the generation of nitrogen vacancies and other structural defects within the g-C_3_N_4_ framework.^15,40^

### Photocatalytic hydrogen evolution performances

**Fig. 4:**
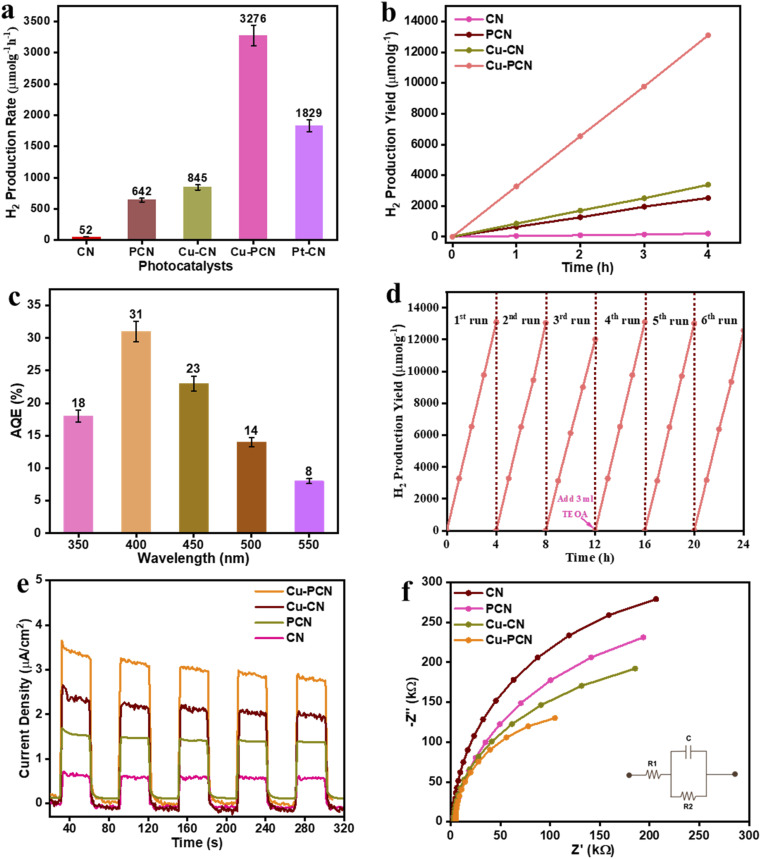
(a) Average photocatalytic H_2_ evolution rates of the as-prepared catalysts, (b) time-dependent H_2_ evolution profiles, (c) wavelength-dependent AQY for H_2_ evolution over Cu-PCN, (d) cycling stability of Cu-PCN during photocatalytic H_2_ production, (e) transient photocurrent response spectra, and (f) EIS plots of CN, PCN, Cu-CN, and Cu-PCN.

The photocatalytic water-splitting performance of all samples was evaluated under visible light without any noble-metal cocatalysts. As shown in Fig. 4a and b, pristine CN exhibits negligible hydrogen evolution, reflecting its poor photocatalytic activity. This is mainly attributed to the scarcity of active sites and the rapid recombination of photogenerated charge carriers. The incorporation of Cu single atoms into g-C_3_N_4_ to form Cu-CN significantly enhances the hydrogen evolution performance, achieving an H_2_ production rate of 845 µmol g^−1^ h^−1^, higher than that of PCN. Furthermore, subsequent phosphating to obtain Cu-PCN results in a remarkable improvement in photocatalytic activity, reaching an H_2_ evolution rate of 3276 µmol g^−1^ h^−1^ over 63 times greater than that of pristine CN. Notably, Cu-PCN also surpasses Pt nanoparticle loaded g-C_3_N_4_ (Pt-CN), demonstrating its superior catalytic efficiency. In addition, compared with previously reported CN-based photocatalysts, Cu-PCN exhibits markedly enhanced water-splitting efficiency (Table S4). The solar energy conversion performance was further evaluated through wavelength-dependent AQY measurements. As illustrated in Fig. 4c, Cu-PCN achieves a maximum AQY of 31% at 400 nm and retains an AQY of 8% at 550 nm, demonstrating its efficient utilization of visible light.

Beyond its high H_2_ evolution activity, Cu-PCN demonstrates excellent stability, showing nearly constant performance over six cycles under visible light (Fig. 4d). A slight decline observed in the third cycle is restored upon adding 3 mL of TEOA, confirming its robust durability. Overall, P-activated single Cu sites enhance light utilization and significantly improve the water-splitting efficiency of CN. Control experiments conducted under identical conditions confirm that hydrogen evolution occurs only when light, catalyst, and TEOA are simultaneously present, with no detectable H_2_ generation in their absence (Fig. S6). Additionally, comparison with various sacrificial electron donors (Fig. S7) shows that TEOA yields the highest hydrogen evolution rate, highlighting its superior effectiveness. Photoelectrochemical (PEC) measurements were performed to elucidate charge-carrier dynamics in the photocatalysts. The transient *I*–*t* curves (Fig. 4e) show that Cu-PCN exhibits a markedly higher photocurrent density than Cu-CN, PCN, and CN, indicating more efficient separation of photogenerated charges. In addition, electrochemical impedance spectroscopy (EIS) results (Fig. 4f) reveal that Cu-PCN possesses the smallest semicircle radius, suggesting lower charge-transfer resistance and faster interfacial kinetics. Collectively, these findings highlight the crucial role of P-modulated single Cu sites in facilitating charge separation and transport for enhanced photocatalytic performance.

**Fig. 5:**
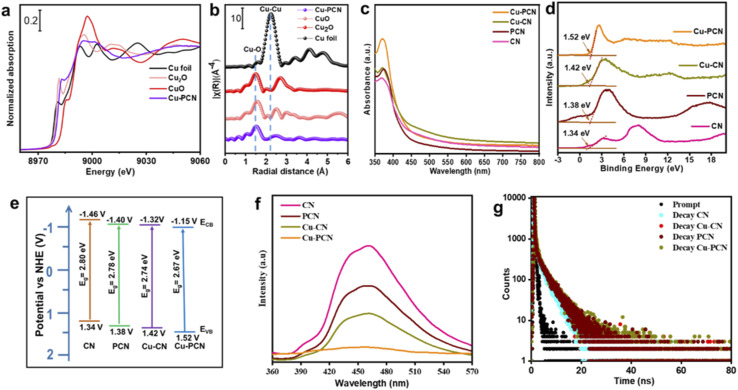
(a) Cu K-edge XANES spectra of Cu foil, Cu_2_O, CuO, and Cu-PCN after catalysis, (b) *k*^3^-weighted Fourier transform EXAFS spectra at the Cu K-edge for the corresponding samples, (c) UV-Vis diffuse reflectance spectra of CN, PCN, Cu-CN, and Cu-PCN; (d) XPS valence band (VB) spectra of CN, PCN, Cu-CN, and Cu-PCN; (e) schematic illustration of the valence band and conduction band positions of CN, PCN, Cu-CN, and Cu-PCN; (f) PL spectra of CN, PCN, Cu-CN, and Cu-PCN; and (g) TRPL decay profiles of CN, PCN, Cu-CN, and Cu-PCN.

The XRD patterns (Fig. S8) and TEM images (Fig. S9) of the catalyst reveal no noticeable changes in the crystal structure or morphology after the reaction, indicating its excellent structural stability. In addition, the Cu K-edge XANES spectra (Fig. 5a) confirm that the oxidation state of the Cu single atoms remains well preserved during the reaction. Consistently, the FT-EXAFS spectra (Fig. 5b) and the *k*-space EXAFS oscillation profiles of Cu-PCN after the reaction (Fig. S10) show no indication of Cu atom aggregation, further evidencing the remarkable atomic dispersion and long-term stability of the Cu single-atom active sites under photocatalytic conditions.

### Optical properties and the photocatalytic mechanism

The band structures and optical properties of the as-synthesized catalysts were investigated using UV-Vis diffuse reflectance spectroscopy (UV-Vis DRS) and valence band X-ray photoelectron spectroscopy (VB-XPS). Fig. 5c presents the UV-Vis diffuse reflectance spectra of the samples. Pristine CN shows weak light absorption, while the introduction of isolated Cu atoms in Cu-CN slightly improves the light-harvesting ability compared with PCN. After thermal phosphidation, Cu-PCN exhibits a pronounced red-shift in the absorption edge along with significantly enhanced visible-light absorption. The corresponding Tauc plots (Fig. S11) show that Cu-PCN has the narrowest bandgap (2.67 eV), compared with CN (2.80 eV), PCN (2.78 eV), and Cu-CN (2.74 eV). These results confirm that structural engineering effectively improves the solar light harvesting efficiency of CN. The valence band (VB) positions of CN, PCN, Cu-CN, and Cu-PCN were determined from VB-XPS spectra (Fig. 5d) to be 1.34, 1.38, 1.42, and 1.52 eV, respectively. The conduction band positions were estimated from the XPS-VB data combined with the band gap values, and are summarized in Fig. 5e.

The charge separation behavior was examined by steady-state photoluminescence (PL) spectroscopy under 370 nm excitation. As shown in Fig. 5f, pristine CN exhibits the strongest emission, indicating severe charge recombination. The PL intensity decreases after P incorporation, suggesting improved charge separation. A further reduction is observed for Cu-CN due to the formation of Cu–N coordination sites that facilitate charge transfer. Notably, Cu-PCN shows the weakest PL emission, confirming its most efficient suppression of electron–hole recombination. Moreover, time-resolved photoluminescence (TRPL) spectroscopy was employed to elucidate the charge carrier kinetics. As depicted in Fig. 5g, Cu-PCN exhibits a slower decay behavior and the longest average lifetime (6.56 ns) relative to CN (4.41 ns), PCN (5.43 ns), and Cu-CN (5.62 ns) (Table S5), indicating more efficient charge separation and suppressed recombination. These results demonstrate that the synergistic interaction between Cu and P effectively enhances light absorption, facilitates charge carrier separation, and prolongs carrier lifetimes.

**Fig. 6:**
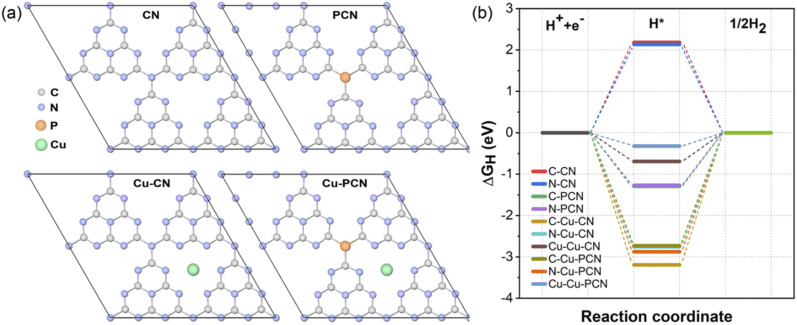
(a) Optimized structures of CN, PCN, Cu-CN and Cu-PCN, and (b) the associated Gibbs free energy diagram for hydrogen adsorption (Δ*G*_H*_).

To further understand the influence of Cu anchored on phosphorus-doped g-C_3_N_4_ in the hydrogen evolution reaction, density functional theory (DFT) calculations were performed. The catalytic models were constructed from tri-*s*-triazine (heptazine)-based g-C_3_N_4_(CN), where the monolayer sheet was obtained by cleaving the bulk structure along the (001) plane. The adopted 2 × 2 × 1 supercell containing 24C and 32 N atoms organized into four heptazine units (C_6_N_7_) interconnected by bridge nitrogen atoms. The phosphorus-doped g-C_3_N_4_ (PCN) was generated by substituting one bridging nitrogen atom that connects adjacent heptazine units with a P atom. For the Cu-anchored CN (Cu-CN) and PCN (Cu-PCN) systems, a Cu atom was placed above the heptazine cavity. The optimized CN, PCN, Cu-CN and Cu-PCN structures are shown in Fig. 6a. The Gibbs free energy of hydrogen adsorption was calculated to evaluate the effect of these structural modifications on the HER activity. Pristine CN exhibits strongly positive values of 2.18 eV for the C site (C-CN) and 2.13 eV for the N site (N-CN), indicating weak hydrogen binding and makes the initial adsorption step energetically unfavourable. In PCN, the Δ*G*_H*_ values decrease to −1.29 eV for the C site (C-PCN) and −1.26 eV for the N site (N-PCN), indicating that P incorporation activates the carbon nitride framework and makes hydrogen adsorption much easier. Although P doping improves the catalytic surface response, the adsorption remains too strong to achieve optimal HER thermodynamics. The influence of Cu becomes evident in Cu-CN as hydrogen adsorption on the Cu centre (Cu-Cu-CN) gives a markedly improved Δ*G*_H*_ of −0.69 eV and serves as the preferred active site, while the adjacent C (C-Cu-CN) and N (N-Cu-CN) sites still indicate strong hydrogen adsorption. As shown in Fig. 6b, Cu-PCN emerges as the most promising system with the Cu site (Cu-Cu-PCN) exhibiting a Δ*G*_H*_ of −0.32 eV, whereas the adjacent C (C-Cu-PCN) and N (N-Cu-PCN) sites remain less favourable due to overly strong binding. Thus, the free energy profiles indicate that the HER activity follows the trend CN < PCN < Cu-CN < Cu-PCN, identifying Cu-PCN as the most effective catalyst. This demonstrates the importance of P doping and Cu anchoring for optimizing hydrogen adsorption, where P modifies the electronic structure of the support and the Cu atom serves as the HER-active site with the most suitable Δ*G*_H*_.

**Fig. 7:**
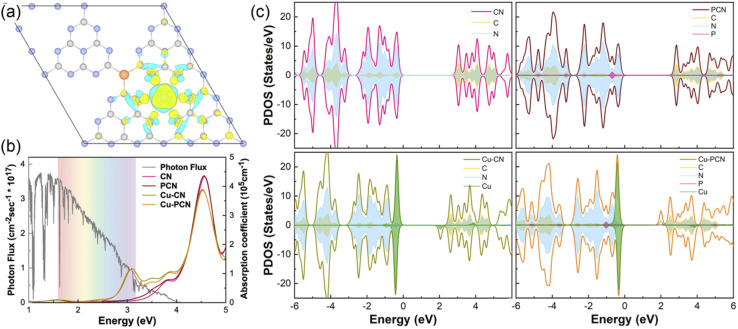
(a) Top view and side view of charge density difference for Cu-PCN. (b) Calculated optical absorption spectra along with the solar photon flux and (c) projected density of states (PDOS) of CN, PCN, Cu-CN and Cu-PCN. The Fermi level is set to zero.

The charge density difference of Cu-PCN reveals strong electronic interaction between the anchored Cu atom and the phosphorus-doped CN support (Fig. 7a). Regions of electron accumulation and depletion are distributed at the Cu–support interface, indicating significant electronic coupling between the Cu active site and the surrounding PCN network. This redistribution polarizes the Cu site and modifies its local electron density, thereby influencing the hydrogen adsorption strength. Bader charge analysis shows that the Cu atom transfers 0.1945 e in Cu-CN and 0.1965 e in Cu-PCN, whereas the P dopant transfers 1.2690 e in PCN and 1.2662 e in Cu-PCN. This behaviour is consistent with the XPS results, where the Cu 2p peak shifts toward lower binding energy in Cu-PCN compared with Cu-CN, indicating that phosphorus primarily modifies the local electronic environment of the Cu active sites. To provide further insight into the origin of the enhanced photocatalytic activity, we analysed the optical and electronic properties. As shown in Fig. 7b, pristine CN exhibits relatively weak absorption in the visible-light region, while phosphorus doping induces only a slight enhancement in the absorption intensity. In contrast, Cu-containing systems exhibit enhanced absorption intensity along with a pronounced red shift toward the visible light region, indicating an improved light harvesting capability. Among all the investigated catalysts, Cu-PCN shows the broadest visible light absorption, demonstrating the effect of Cu anchoring and P doping in extending the photo response toward lower photon energies. The enhanced visible light absorption enables more efficient utilization of solar energy and promotes the generation of a larger population of photogenerated charge carriers,^41,42^ which is beneficial for photocatalytic hydrogen evolution. As shown in Fig. 7c, the projected density of states further reveals that pristine CN exhibits semiconducting electronic structure with well separated valence and conduction-band states. Phosphorus doping slightly modifies the electronic states near the band edges, whereas Cu anchoring introduces additional Cu states in the vicinity of the Fermi level for Cu-CN and Cu-PCN. These additional electronic states reduce the energy required for electronic excitation and provide accessible electronic channels that facilitate charge transport during the photocatalytic process. The calculated electronic band structures show that pristine CN possesses a band gap of 2.81 eV (Fig. S12), while phosphorus doping slightly narrows the band gap of PCN to 2.57 eV. The introduction of Cu further decreases the band gap to 1.85 eV for Cu-CN and 1.76 eV for Cu-PCN. The significant reduction in band gap demonstrates that Cu anchoring lowers the excitation energy required for electron transition from the valence band to the conduction band by introducing additional electronic states. Also, the reduced band gap of Cu-PCN is consistent with its enhanced visible light absorption, demonstrating that the interaction between Cu and P improves the light harvesting capability of the catalyst.

**Fig. 8:**
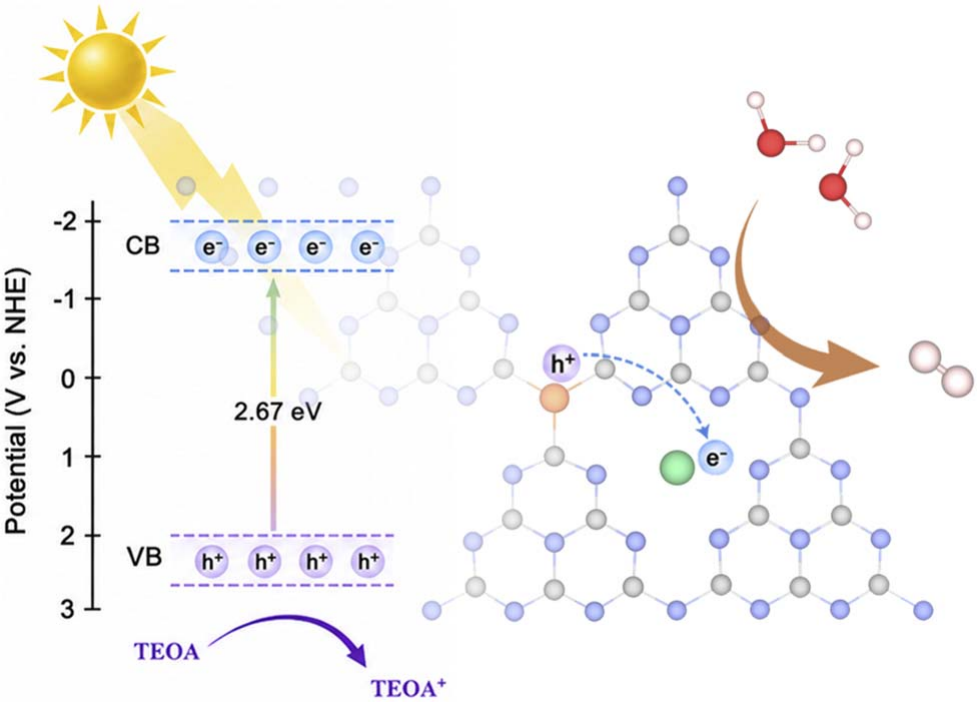
Schematic illustration of the proposed photocatalytic hydrogen evolution mechanism over the Cu-PCN photocatalyst.

The photocatalytic HER mechanism over Cu-PCN proceeds through the synergistic interplay of its constituent components. The incorporation of phosphorus into the carbon nitride framework induces redistribution of the electron density and generates Lewis acidic sites, which effectively spatially separate redox centers and promote directional charge migration. Under visible-light irradiation, photogenerated electrons are preferentially transferred to the Cu single-atom sites, where they function as highly active catalytic centers for hydrogen evolution. Concurrently, TEOA serves as a sacrificial hole scavenger, rapidly consuming photogenerated holes and suppressing charge recombination (Fig. 8). Collectively, the electron-modulating effect of phosphorus, the catalytic functionality of Cu single atoms, and the efficient hole extraction by TEOA work cooperatively to maximize charge utilization, thereby markedly enhancing the photocatalytic hydrogen evolution performance.

## Conclusion

In this study, we introduce a simple effective approach to activate single-atom Cu catalytic centers by modulating their electronic configuration through phosphorus incorporation. The optimized Cu-PCN catalyst delivers an outstanding hydrogen evolution rate of 3276 µmol g^−1^ h^−1^ with an apparent quantum yield of 31% at 400 nm, along with excellent stability, without the need for noble-metal cocatalysts. Density functional theory results reveal that phosphorus incorporation activates the carbon nitride framework, while the Cu site serves as the primary catalytic center with a near-optimal hydrogen adsorption free energy of Δ*G*_H*_ of −0.32 eV. The synergistic interaction between Cu and P induces charge redistribution, enhances electronic coupling, and lowers the reaction energy barrier, thereby accelerating interfacial electron transfer and hydrogen generation kinetics. Overall, this work provides clear atomic-level insights into designing asymmetric and highly efficient photocatalysts for sustainable solar hydrogen production.

## Author contributions

H. I. conducted most of the experimental work, including material synthesis, characterization, data analysis, and manuscript drafting. B. J. contributed to data analysis. S. M. and S. S. helped to perform the photocatalytic experiments. B. B. S. carried out the XAS analysis and manuscript revision. B. M. A. performed the theoretical analysis. U. P. conceived the research, supervised the study, revised the manuscript, and secured funding. All authors contributed to the manuscript preparation and approved the final version.

## Conflicts of interest

The authors declare no conflict of interest.

## Supplementary Material

SC-OLF-D6SC04795E-s001

## Data Availability

The data that support the findings of this study are available within the article and supplementary information (SI). Supplementary information is available. See DOI: https://doi.org/10.1039/d6sc04795e.

## References

[cit1] Islam H., Jaksani B., Iqbal A., Varangane S., Annadata H. V., Ghosh B., Sarma B. B., Thapa R., Pal U. (2025). Atomically Dispersed Cu-Ni Dual-Metal Sites on g-C_3_N_4_ for Synergistic Enhancement of Photocatalytic Hydrogen Evolution. ACS Appl. Energy Mater..

[cit2] Sk S., Islam H., Abraham B. M., Mondal I., Pal U. (2025). Defects in MOFs for photocatalytic water reduction to hydrogen generation: from fundamental understanding to state-of-art materials. Small Methods.

[cit3] Shen J., Luo C., Qiao S., Chen Y., Tang Y., Xu J., Fu K., Yuan D., Tang H., Zhang H., Liu C. (2023). Single-Atom Cu Channel and N-Vacancy Engineering Enables Efficient Charge Separation and Transfer between C_3_N_4_ Interlayers for Boosting Photocatalytic Hydrogen Production. ACS Catal..

[cit4] Jaksani B., Islam H., Annadata H. V., Ghosh B., Abraham M. B., Sudarshan K., Mahatha S. K., Pal U., Mondal I. (2026). Role of catalyst transformations, defect reconstruction, and seawater splitting under a broad solar spectrum using a MOF-derived (Cu)TiO_2_ model system. J. Mater. Chem. A.

[cit5] Dharmarajan N. P., Vidyasagar D., Yang J.-H., Talapaneni S. N., Lee J., Ramadass K., Singh G., Fawaz M., Kumar P., Vinu A. (2024). Bio-Inspired Supramolecular Self-Assembled Carbon NitrideNanostructures for Photocatalytic Water Splitting. Adv. Mater..

[cit6] Wu G., Wang Q., Ren Q., Mo Z., Xu H. (2025). Molecular Structure Engineering of Graphitic Carbon Nitride for Photocatalytic Hydrogen Evolution: Recent Advances and Perspectives. Small.

[cit7] Jiang T., Wang Z., Wei G., Wu S., Huang L., Li D., Ruan X., Liu Y., Jiang C., Ren F. (2024). Defective High-Crystallinity g-C_3_N_4_ Heterostructures by Double-End Modulation for Photocatalysis. ACS Energy Lett..

[cit8] Xu R., Xu B., You X., Shao D., Gao G., Li F., Wang X.-L., Yao Y.-F. (2023). Preparation of single-atom palladium catalysts with high photocatalytic hydrogen production performance by means of photochemical reactions conducted with frozen precursor solutions. J. Mater. Chem. A.

[cit9] Zhu A., Cao Y., Zhao N., Jin Y., Li Y., Yang L., Zhang C., Gao Y., Zhang Z., Zhang Y., Xie W. (2024). Geminal Synergy in Pt-Co Dual-Atom Catalysts: From Synthesis to Photocatalytic Hydrogen Production. J. Am. Chem. Soc..

[cit10] Wu G., Zhang W., Mo Z., Zhao X., Sun P., Wang Q., Yan P., She X., Xu H. (2025). S-Scheme Homojunction Carbon Nitride for Photocatalytic Overall Water Splitting. ACS Catal..

[cit11] Ren M., Zhang X., Liu Y., Yang G., Qin L., Meng J., Guo Y., Yang Y. (2022). Interlayer palladium-single-atom-coordinated cyano-group-rich graphitic carbon nitride for enhanced photocatalytic hydrogen production performance. ACS Catal..

[cit12] Islam H., Iqbal A., Jaksani B., Kshirsagar S. D., Sudarshan K., Thapa R., Ahmadipour M., Pal U. (2026). Modulation of Sulfur Vacancies in MOF-Derived 3D Hollow ZnCo_2_S_4_ Polyhedra on 2D g-C_3_N_4_ Nanosheets for Enhanced Photocatalytic
Hydrogen Evolution. ACS Appl. Energy Mater..

[cit13] Majdoub M., Anfar Z., Amedlous A. (2020). Emerging Chemical Functionalization of g-C_3_N_4_: Covalent/Noncovalent Modifications and Applications. ACS Nano.

[cit14] Zhong T., Huang W., Yao Z., Long X., Qu W., Zhao H., Tian S., Shu D., He C. (2024). Engineering of Graphitic Carbon Nitride (g-C_3_N_4_) Based Photocatalysts for Atmospheric Protection: Modification Strategies, Recent Progress, and Application Challenges. Small.

[cit15] Shen J., Luo C., Qiao S., Chen Y., Tang Y., Xu J., Fu K., Yuan D., Tang H., Zhang H. (2023). Single-Atom Cu Channel and N-Vacancy Engineering Enables Efficient Charge Separation and Transfer between C_3_N_4_ Interlayers for Boosting Photocatalytic Hydrogen Production. ACS Catal..

[cit16] Chen W., Yu M., Liu S., Zhang C., Jiang S., Duan G. (2024). Recent Progress of Ru Single-Atom Catalyst: Synthesis, Modification, and Energetic Applications. Adv. Funct. Mater..

[cit17] Zhao Q.-P., Shi W.-X., Zhang J., Tian Z.-Y., Zhang Z.-M., Zhang P., Wang Y., Qiao S.-Z., Lu T.-B. (2024). Photo-induced synthesis of heteronuclear dual-atom catalysts. Nat. Synth..

[cit18] Islam H., Mishra G., Biswas B., Jaksani B., Bhatt H., Das S., Ghosh H. N., Mahatha S. K., Ghosh K., Sarma B. B., Ghosh S., Pal U. (2026). Atomic-Level Synergy of Dual Single-Atom Catalysts for Photocatalytic Hydrogen Evolution Reaction. Small.

[cit19] Liu Y., Sun Y., Zhao E., Yang W., Lin J., Zhong Q., Qi H., Deng A., Yang S., Zhang H., He H., Liu S., Chen Z., Wang S. (2023). Atomically Dispersed Silver-Cobalt Dual-Metal Sites Synergistically Promoting Photocatalytic Hydrogen Evolution. Adv. Funct. Mater..

[cit20] Lazaar N., Wu S., Qin S., Hamrouni A., Bikash Sarma B., Doronkin D. E., Denisov N., Lachheb H., Schmuki P. (2025). Single-Atom Catalysts on C_3_N_4_: Minimizing Single Atom Pt Loading for Maximized Photocatalytic Hydrogen Production Efficiency. Angew. Chem., Int. Ed..

[cit21] Yadav D. K., Latiyan S., Devan R. S., Urkude R. R., Rajput P., Singh A., Deka S. (2025). Breaking Barriers: Synergistic Interactions Between Pt Single Atoms and Nitrogen-Rich g-C_3_N_4_ for Maximized Photocatalytic Hydrogen Production. Small.

[cit22] Hussien M. K., Sabbah A., Qorbani M., Putikam R., Kholimatussadiah S., Tzou D.-L. M., Elsayed M. H., Lu Y.-J., Wang Y.-Y., Lee X.-H., Lin T.-Y., Thang N. Q., Wu H.-L., Haw S.-C., Wu K. C.-W., Lin M.-C., Chen K.-H., Chen L.-C. (2024). Constructing B-N-P Bonds in Ultrathin Holey g-C_3_N_4_ for Regulating the Local Chemical Environment in Photocatalytic CO_2_ Reduction to CO. Small.

[cit23] Xie W., Li K., Liu X.-H., Zhang X., Huang H. (2023). P-Mediated Cu-N_4_ Sites in Carbon Nitride Realizing CO_2_ Photoreduction to C_2_H_4_ with Selectivity Modulation. Adv. Mater..

[cit24] Cheng X., Bi Y., Liu X., Ji L., Feng C., Gao S., Li H., Shang N., Gao W., Meng T., Wang C., Wang L. (2025). Unraveling the Microstructure-Property Relationship of Fe Single-Atoms *via* Introducing Asymmetric P-Coordination for Photocatalytic Hydrogen Evolution. Adv. Funct. Mater..

[cit25] Li T., Yang Z., Xu Q., Sun T., Ma X., Zheng T., Wang Y., Wang Z., Li Q., Yu Q., Jiang J. (2025). Asymmetrical Degree Engineered Carbon DioxidePhotoreduction for Single Atomic Co Sites on PolymericCarbon Nitride. Adv. Funct. Mater..

[cit26] Ya Z., Li M., Xu D., Wang H., Zhang S. (2025). Photoreforming of Plastic Waste: Connecting the Dots for Green and Efficient Upcycling Systems within a Circular Economy. ACS Nano.

[cit27] Zhou T., Wei H., Xiao B., Lv T., Duan L., Lu Q., Zhang J., Zhang Y., Liu Q. (2023). Anchored Cu single atoms on porous gC_3_N_4_ for superior photocatalytic H_2_ evolution from water splitting. RSC Adv..

[cit28] Li B., Si Y., Fang Q., Shi Y., Huang W.-Q., Hu W., Pan A., Fan X., Huang G.-F. (2020). Hierarchical self-assembly of well-defined louver-like P-doped carbon nitride nanowire arrays with highly efficient hydrogen evolution. Nano–Micro Lett..

[cit29] He T., Reuter K., Du A. (2020). Atomically dispersed asymmetric Cu–B pair on 2D carbon nitride synergistically boosts the conversion of CO into C_2_ products. J. Mater. Chem. A.

[cit30] Li B., Zheng H., Zhou T., Lu Q., Chen M., Sun H., Zhang Y., Zhang Y., Li D., Zi B., Zhang M., Zhang J., Zhao J., He T., Zhu Z., Zhang G., Liu Q. (2025). Asymmetric coordination enhances the synergy of Pt species dual active sites for efficient photocatalytic H_2_ evolution. Nat. Commun..

[cit31] Shi H., Wang H., Zhou Y., Li J., Zhai P., Li X., Gurzadyan G. G., Hou J., Yang H., Guo X. (2022). Atomically Dispersed Indium-Copper Dual-Metal Active Sites Promoting C−C Coupling for CO_2_ Photoreduction to Ethanol. Angew. Chem., Int. Ed..

[cit32] Lian Z., Luo D., Yang J., Yang Y., Tang S., Li H., Zhang D., Li H. (2025). Spatial Decoupling of Adsorption and Transformation Sites on Ag-Cu Dual-Single-Atom Catalysts for Highly Selective Photocatalytic Nitrate-to-Ammonia Reduction. Angew. Chem., Int. Ed..

[cit33] Bao X., Li M., Xie Y., Luo X., Tong F., Liang X., Xiao D., Wang Z. (2026). Constructing the asymmetrically coordinated Pt single atoms by doping g-C_3_N_4_ support for enhanced photocatalytic plastic reforming for H_2_ evolution. J. Colloid Interface Sci..

[cit34] Wang Y. T., Lin H. Y., Chen Y. C., Lin Y. G., Wu J. M. (2024). Piezo-Flexocatalysis of Single-Atom Pt-Loaded Graphitic Carbon Nitride. Small Methods.

[cit35] Chava R. K., Kang M. (2023). Engineering morphology and nitrogen content on graphitic carbon nitrides for efficient solar to hydrogen conversion reaction. Appl. Surf. Sci..

[cit36] Shao W., Yu M., Xu X., Han X., Chen Y., Han J., Wu G., Xing W. (2024). Design of a Single-Atom In–N_3_–S Site to Modulate Exciton Behavior in Carbon Nitride for Enhanced Photocatalytic Performance. Small.

[cit37] Chava R. K., Raja A., Panneer Selvam S., Kamalakannan S., Kang M. (2026). Mechanistic insights into the formation of N-vacancies at sp^2^-hybridized sites in non-metal doped gC_3_N_4_ for solar-to-H_2_ conversion and environmental remediation. J. Mater. Chem. A.

[cit38] Ding J., Xu W., Wan H., Yuan D., Chen C., Wang L., Guan G., Dai W. L. (2018). Nitrogen Vacancy Engineered Graphitic C_3_N_4_-Based Polymers for Photocatalytic Oxidation of Aromatic Alcohols to Aldehydes. Appl. Catal., B.

[cit39] Sarma B. B., Maurer F., Doronkin D. E., Grunwaldt J.-D. (2023). Design of Single-Atom Catalysts and Tracking Their Fate Using *Operando* and Advanced X-ray Spectroscopic Tools. Chem. Rev..

[cit40] Yang M., Mei J., Ren Y., Cui J., Liang S., Sun S. (2023). Long-range electron synergy over Pt_1_-Co_1_/CN bimetallic single-atom catalyst in enhancing charge separation for photocatalytic hydrogen production. J. Energy Chem..

[cit41] Long C., Wan K., Qiu X., Zhang X., Han J., An P., Yang Z., Li X., Guo J., Shi X., Wang H., Tang Z., Liu S. (2022). Single site catalyst with enzyme-mimic micro-environment for electroreduction of CO_2_. Nano Res..

[cit42] He T., Tang C., Puente Santiago A. R., Luque R., Pan H., Du A. (2021). Tuning CO Binding Strength *via* Engineering the Copper/Borophene Interface for Highly Efficient Conversion of CO into Ethanol. J. Mater. Chem. A.

