# Supplementary material for: Asymmetric atomic-level engineering of phosphorus-modified Cu single-atom coordination centers on carbon nitride for enhanced photocatalytic hydrogen evolution
Source: Chem Sci. 2026 Jul 30;17(37):17953–63. doi: 10.1039/d6sc04795e (PMC13448454; doi:10.1039/d6sc04795e)
Supplement: Supplementary file 1 [file SC-017-D6SC04795E-s001.pdf]

Supplementary Information (SI) for Chemical Science.
This journal is © The Royal Society of Chemistry 2026

Supporting Information

Asymmetric atomic-level engineering of phosphorus-modified Cu single-atom coordination centers on carbon nitride for enhanced photocatalytic hydrogen evolution.

Hafijul Islam, [a, b] Bhavya Jaksani, [a, b] Saad Mehmood, [a, b] Sukanya Saha, [a, b] Bidyut Bikash Sarma, [c] B. Moses Abraham, [d, e] Ujjwal Pal*. [a, b]

[a]Department of Energy & Environmental Engineering, CSIR-Indian Institute of Chemical Technology, Hyderabad, Telangana 500007, India.

[b]Academy of Scientific and Innovative Research (AcSIR), Ghaziabad 201002, India.

[c] University of Toulouse, Toulouse INP, CNRS, LCC, Toulouse, France.

[d] A.J. Drexel Nanomaterials Institute, and Department of Materials Science and Engineering Drexel University, Philadelphia 19104, USA.

[e] Department of Chemical Engineering, Indian Institute of Technology Kanpur, Kanpur-208016, India.

Correspondence: Ujjwal Pal (upal03@gmail.com, ujjwalpal@iict.res.in)

1. EXPERIMENTAL SECTION

1.1. Chemical Reagents

Melamine (MA, 98%), Cyanuric acid (CA, 98%), Citric acid (CTA, 99.5%), Cu $(NO_3)_2.3H_2O$, 99%), Sodium hypophosphite (NaH_2PO_2, 99%), Sodium Sulfate (Na_2SO_4, 99.0%), Triethanolamine (TEOA, 99%), Nafion solution and ethanol were purchased from Sigma-Aldrich and used as received without any additional purification.

1.2. Preparation of Photocatalysts

1.2.1. Preparation of Cu-CN and CN

Atomically dispersed Cu-CN was prepared via a thermal pyrolysis polymerization route. In a typical synthesis, melamine (MA, 10 mmol) and cyanuric acid (CA, 9.5 mmol) were individually dissolved in 30 mL deionized water at 85 °C under stirring until clear solutions were obtained, denoted as solutions A and B, respectively. Citric acid (CTA, 2.5 mmol) and Cu $(NO_3)_2{\cdot}3H_2O$ (0.25 mmol) were dissolved in 10 mL deionized water to form solution C, which was then added to solution B and stirred for 10 min. The resulting mixture was

subsequently added dropwise into solution A and further stirred at room temperature for 4 h to generate supramolecular precursors. The product was collected by centrifugation, washed three times with deionized water and ethanol, and dried under vacuum at 60 °C for 12 h. The dried solid was ground and calcined in a tube furnace at 600 °C for 2 h under N_2 flow with a heating rate of 5 °C/min to obtain Cu-CN. For comparison, pristine CN was synthesized under the same conditions without addition of metal precursors.

1.2.2. Preparation of Cu-PCN and PCN

Cu-PCN was synthesized via a thermal phosphidation process using sodium hypophosphite (NaH_2PO_2) as the phosphorus source. In a typical procedure, 50 mg of Cu-CN was thoroughly mixed and ground with 300 mg of NaH_2PO_2 in a mortar. The mixture was then annealed at 300 °C for 2 h under a nitrogen atmosphere with a heating rate of 2 °C min^{-1}. The resulting powder was washed several times with ultrapure water and ethanol to remove soluble impurities and subsequently dried at 60 °C. For comparison, PCN was prepared following the same phosphidation process, using pristine CN instead of Cu-CN.

1.2.3. Preparation of Pt nanoparticle doped CN (Pt-CN)

Pt-CN was synthesized via a facile photodeposition method. In a typical procedure, 100 mg of pristine CN was dispersed in 60 mL of a 10 vol% triethanolamine aqueous solution using ultrasonic treatment for 20 min. Subsequently, 420 μL of H_2PtCl_6 solution (12.2 mM) was added dropwise under continuous stirring and kept in the dark for 20 min. The suspension was then sealed, and high-purity Ar gas was bubbled through for 20 min to remove dissolved oxygen. Afterwards, the mixture was irradiated with a 300 W Xe lamp at 20 °C for 1 h to reduce H_2PtCl_6 to metallic Pt. Finally, the product was collected, thoroughly washed with deionized water, and dried at 60 °C to obtain the Pt-CN photocatalyst.

1.3. Photocatalytic Experiment Condition

Photocatalytic hydrogen evolution tests were conducted under simulated solar irradiation using a 420 W Xe lamp fitted with a 400 nm band-pass filter in a 100 mL quartz photoreactor. Typically, 10 mg of photocatalyst was dispersed in 50 mL of an aqueous 10% TEOA solution. The suspension was sealed in a quartz container, and dissolved gases were removed by vacuum treatment for 15 min, followed by nitrogen purging for 30 min to create an inert atmosphere. The reactor was then positioned in front of the light source and continuously stirred during irradiation. Gas samples were collected at regular intervals with a 100 μL airtight syringe and analyzed using a PerkinElmer gas chromatograph (Clarus 590) equipped with a thermal conductivity detector (TCD), with nitrogen serving as the carrier gas. The pH of water was 7,

the medium with 10 vol% TEOA was 10.2, and the medium containing water, TEOA, and the catalyst was 10.3.

For sacrificial reagent test, the 10.0 vol% TEOA solution was replaced by, 10.0 vol% methanol, 10.0 vol% ethanol, 10.0 vol% TEA, 10.0 vol% lactic acid, 0.1 M (10.0 vol%) glucose solution, and 0.1M (10.0 vol%) ascorbic acid solution.

1.4. Material Characterisation

The structural phase analysis of the as-synthesized photocatalysts was conducted using powder X-ray diffraction (XRD) patterns on a Bruker AXS diffractometer (D8 Advance) at a generator voltage of 40 kV and a current of 30 mA, employing Cu-Kα_1 radiation (λ = 1.5406 Å). The sample was scanned over a 2θ range of 5-80 ° with a scan rate of 1 s/step. X-ray photoelectron spectroscopy (XPS) was performed using a Kratos Axis 165 analytical instrument with Mg Kα radiation, maintaining a spectrometer pressure of approximately10^{-9}. The structural morphology of the photocatalysts was examined by using transmission electron microscopy (TEM) images were obtained using a JEOL 2010EX TEM instrument equipped with a high-resolution objective-lens pole piece at an acceleration voltage of 200 kV, fitted with a CCD camera**.** High angle annular dark-field scanning transmission electron microscopy (HAADF-STEM) imaging was performed employing an aberration corrected Thermo Fisher Scientific Spectra 200 operating at 200 keV, and featuring an ultra-high brightness cold field emission gun (X-CFEG). The optical properties were characterized by using UV-Vis diffuse reflectance spectroscopy (DRS) Perkin Elmer Lambda 750 instrument using $BaSO_4$ as a reference. The sample has been placed in the sample holder for the measurement and the light is allowed to pass through the sample which leads to the absorption of the light and the light transmitted by the sample has been recorded. The transmitted light was recorded for analysis. Photoluminescence (PL) spectra were recorded using a Fluorolog-3 spectrofluorometer (Spex model, Jobin Yvon) at their respective excitation wavelengths (λex). Lifetime measurements were performed using a Horiba DeltaFlex-01 TDM-850 emission monochromator in solid-state. A 340 nm laser was used for excitation, with a bandpass filter set at 6 nm.

1.5. Photo-Electrochemical investigations

Photoelectrochemical measurements were performed at room temperature using a CHI6005E electrochemical workstation (CH Instruments Inc., USA) equipped with a Potentiostat. A conventional three-electrode was employed, consisting of an ITO film coated with the photocatalyst as the working electrode, a platinum wire as the counter electrode, and an Ag/AgCl electrode as the reference. The artificial solar simulator of AM 1.5 G illuminator (100

mW cm^{-2}) was used as the light source during the measurement. The electrochemical cell was constructed from Pyrex glass, and 0.25 M Na_2SO_4 aqueous solution served as the electrolyte. Electrochemical impedance spectroscopy (EIS) was used to analyzed the interfacial charge-transfer behavior between the electrode and electrolyte. The Nyquist plots were recorded under light illumination at an open-circuit voltage of 0.10 V, over a frequency range of 1 Hz to 1 MHz with an applied AC amplitude of 5 mV. The diameter of the semicircle in the Nyquist plot reflects the charge-transfer resistance, with smaller diameters indicating more efficient electron transport.

Electrode Preparation: To prepare the working electrode, 4 mg of each as-synthesized photocatalyst was dispersed into a suspension that contained 0.5 ml ethanol and 10 µL Nafion by 30 min of ultrasonication. The as-prepared solution was dropped on the surface of Indium tin oxide (ITO) film of 2 × 2 cm^2 surface area to achieve uniform coverage and then dried in air at room temperature.

1.6. Kubelka-Munk theory

Tauc introduced a method for estimating the band gap energy of amorphous and other semiconductor materials through the analysis of optical absorption spectra (DRS). This methodology was later refined by Davis and Mott. The Tauc method is based on the assumption that the energy-dependent absorption coefficient (α) can be represented by a specific equation. Tauc plot is utilized to determine the optical bandgap for disordered, amorphous, and crystalline semiconductor photocatalysts. Typically, a Tauc plot displays photon energy (hv) on the x-axis and $(\alpha h\nu)^{1/2}$ on the y-axis, where α represents the absorption coefficient of the material. The bandgaps of CN, PCN, Cu-CN and Cu-PCN are obtained via the equation below.

$$\alpha h\nu = A\,(h\nu - E_g)^{n/2}$$

α, ν, E_g, and A are absorption coefficient, light frequency, bandgap, and constant, respectively. n is 1 for direct transition type semiconductor of CN. The bandgaps of CN, PCN, Cu-CN and Cu-PCN determined 2.80 eV, 2.78 eV, 2.74 eV and 2.67 eV.

1.7. Instrument and Experiment Details for X-ray Absorption Spectroscopy (XAS)

X-ray absorption spectra at the Cu K-edge (8,979 eV) was collected at the P65 beamline of the Deutschers Elektronen-Synchrotron (DESY), Hamburg. To scan the incident energy, a Si (111) and Si (311) double crystal monochromator was used. For measuring the samples and references, pellets diluted with cellulose were used, and the measurements were conducted in transmission or fluorescence mode. The processing of the XAS results (data reduction) was performed by using the Demeter software package. Data treatment (alignment, normalization)

has been performed using the Athena code (version 0.9.26). For all fits, k value up to 12 $Å^{-1}$ and radial distance (R) between 1-2.2 Å were considered.

1.8. Apparent quantum yield (AQY) calculation for H_2 production

The apparent quantum yield (AQY) of Cu-PCN for photocatalytic hydrogen evolution at different wavelength (350 nm, 400 nm, 450nm, 500nm, 550 nm) are calculated. A 420 W Xe lamp served as the light source. The experimental conditions were carefully considered, and an optical power meter (Newport, Model: 842-PE) was utilized for the measurements. The average incident irradiation was measured at 139.8 mW/cm^2, based on the Newport (Oriel) lamp with an irradiation area of 12.62 cm^2. The hydrogen production rate for Cu-PCN was determined to be 3276 μmol g^{-1} h^{-1}. The number of incident photons ($N_{photons}$) and AQY (%) were calculated using the equations provided below:

$$N_{photon} = \frac{\mathrm{P\lambda t}}{\mathrm{hc}}$$

Here, P = power of light (139.8 mW/cm^2 = 0.139 J s^{-1} cm^{-2}) over a specific area of 12.62 cm^2, λ = light wavelength (400 nm), t = irradiation time (h), h = Planck's constant (6.626 * 10^{-34} J s), and c = velocity of light (3 *10^8 m s^{-1}).

The number of incident photon in 1h,

$$N_{photon} = \frac{0.139 \times 12.62 \times 400 \times 10^{-9} \times 3600}{6.626 \times 10^{-34} \times 3 \times 10^{8}} = 0.127 \times 10^{23}$$

$$\mathrm{AQY} = \frac{(2 \times \text{ the no of } \mathrm{H_2} \text{ molecules produced})}{\text{The no. of incident photons}} \times 100\ \%$$

$$\mathrm{AQY} = \frac{2 \times 3.276 \times 10^{-3} \times 6.02 \times 10^{23}}{0.127 \times 10^{23}} \times 100\ \% = 31\%$$

1.9. Computational details

Electronic structure calculations were carried out within a first-principles framework using the Vienna Ab initio Simulation Package.[1, 2] The interaction between ionic cores and valence electrons was represented through the projector augmented wave formalism,[3] while structural relaxation was performed until the residual force on each atom was smaller than 0.01 eV $Å^{-1}$. The exchange-correlation energy was described using a generalized gradient approximation in the Perdew-Burke-Ernzerhof form ,[4] where the electronic convergence was achieved when the

total energy change between consecutive self-consistent steps was less than 10^{-5} eV. A kinetic energy cutoff of 520 eV was selected for plane-wave expansion. The first Brillouin zone was sampled using a Monkhorst-Pack k-point mesh of 5 × 5 × 1 [5]. A vacuum spacing of more than 10 Å was applied along the out-of-plane direction to prevent interactions between periodically repeated layers. Long-range van der Waals interactions were accounted for using the Grimme DFT-D3 scheme to capture weak intermolecular interactions [6]. Four model systems were considered, namely pristine graphitic carbon nitride (CN, corresponding to g-C_3N_4), P-doped g-C_3N_4 (PCN), Cu-anchored g-C_3N_4 (Cu-CN), and Cu single atoms anchored on P-doped g-C_3N_4 (Cu-PCN). The Gibbs free energy of hydrogen adsorption, ΔG_{H*} was used as the descriptor for HER activity and was obtained from: $\Delta G_{H*} = \Delta E_{H*} + \Delta E_{ZPE} - T\Delta S$; where ΔE_{H*} denotes the electronic adsorption energy. The zero-point energy correction and entropy contribution were obtained from vibrational frequency calculations according to $\Delta E_{ZPE} = ZPE_{H*} - \frac{1}{2} ZPE_{H_2}$ $and\ T\Delta S = TS_{H*} - \frac{1}{2} TS_{H_2}$; where ZPE_{H*} and TS_{H*} correspond to adsorbed hydrogen species[7], while ZPE_{H_2} and TS_{H_2} denote the zero-point energy and entropy of gas phase H_2, respectively. The electronic term was obtained from the total energies of the adsorbed and non-adsorbed systems according to $\Delta E_{H*} = E_{slab+H} - E_{slab} - 1/2E_{H2}$; where E_{slab+H} is the total energy of the substrate after hydrogen adsorption, E_{slab} corresponds to the clean catalyst surface, and E_{H2} is the energy of an isolated hydrogen molecule To understand the electronic interaction introduced by Cu anchoring, charge redistribution was analysed for the Cu-PCN system using the differential charge density: $\Delta\rho = \rho_{Cu\text{-}PCN} - \rho_{PCN} - \rho_{Cu}$; where $\rho_{Cu\text{-}PCN}$ is the charge density of the fully combined structure, ρ_{PCN} is that of the supporting sheet, and ρ_{Cu} is that of the isolated Cu atom.

2. RESULTS AND DISCUSSION

2.1. Supporting Figures

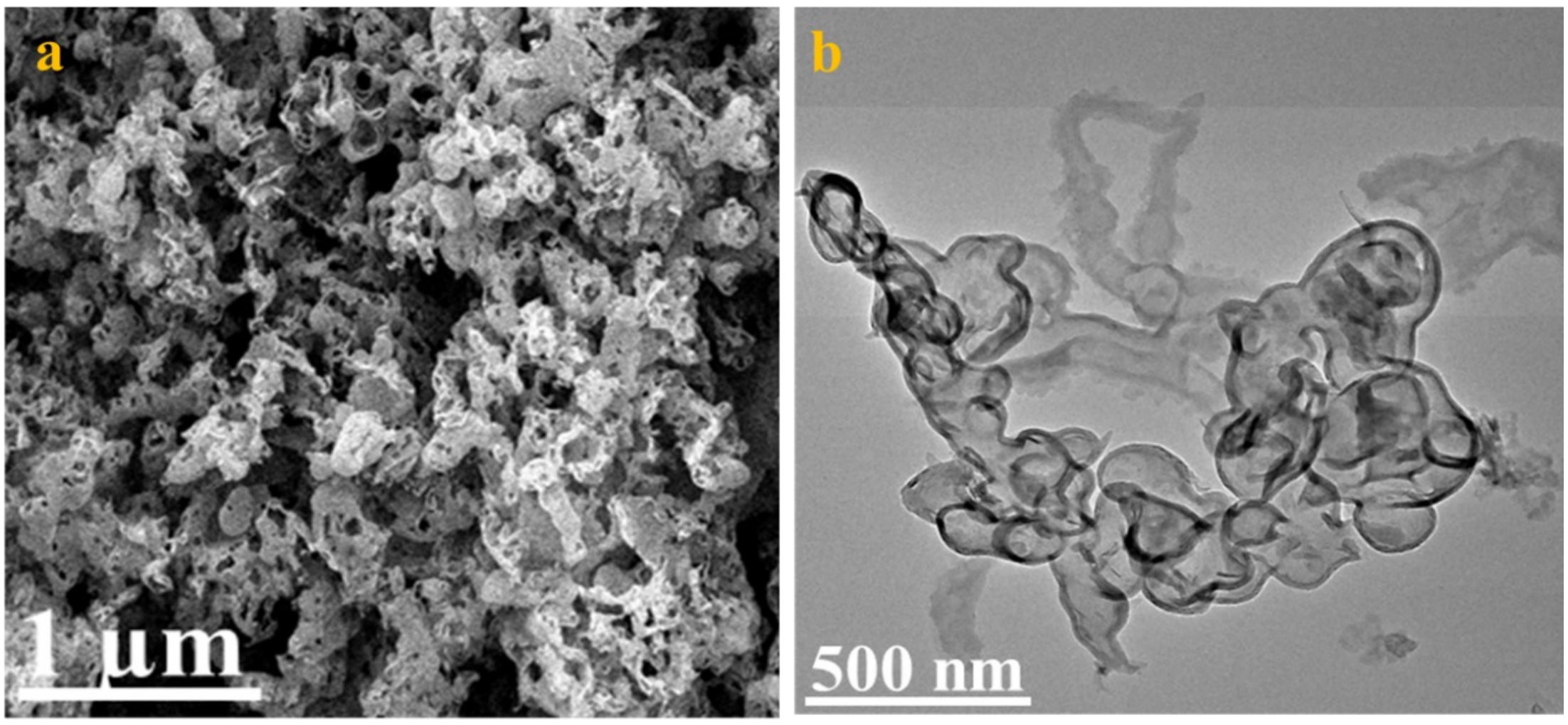

Fig S1. (a) SEM and (b)TEM image of Cu-CN

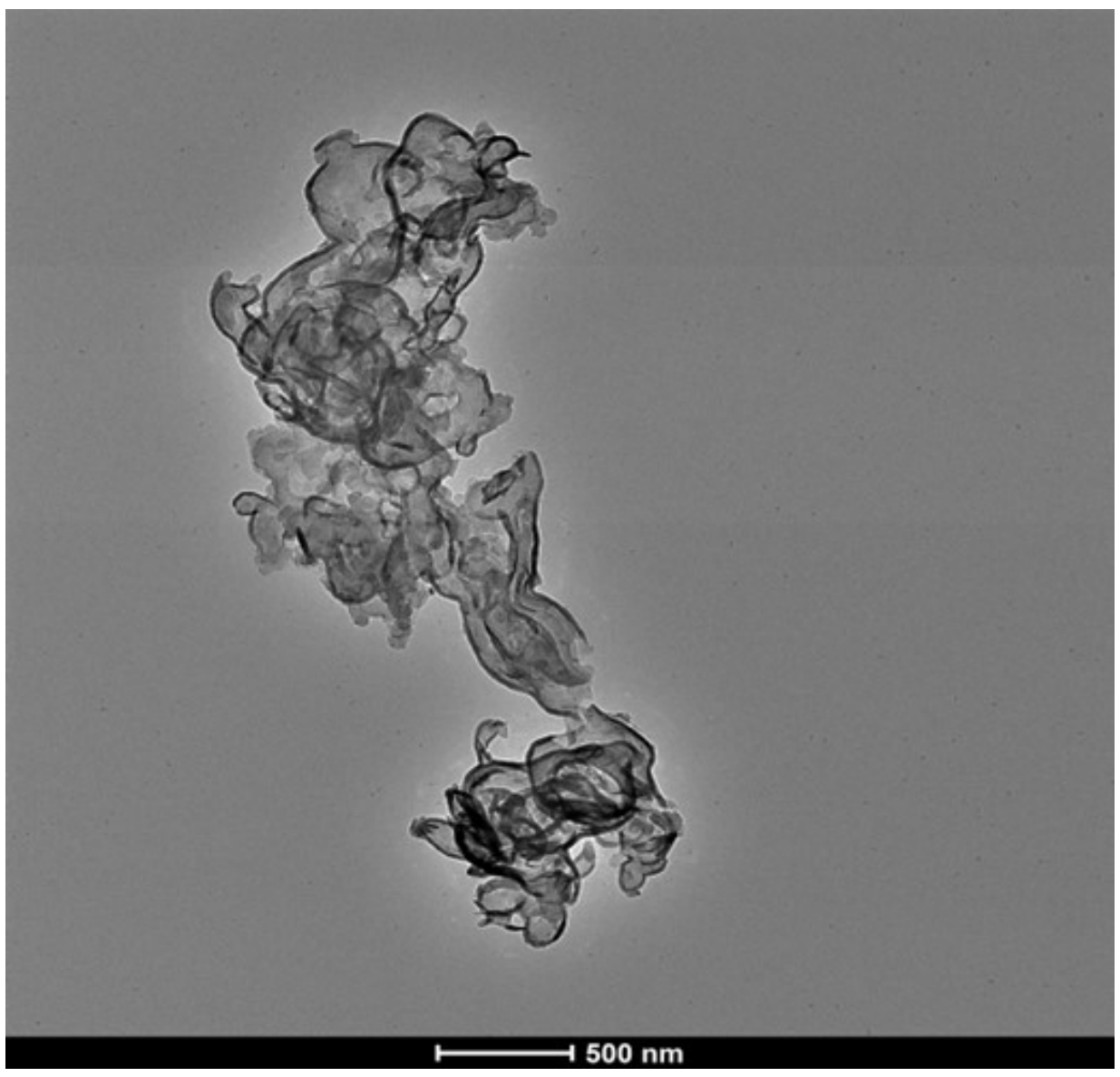

Fig S2. TEM image of CN

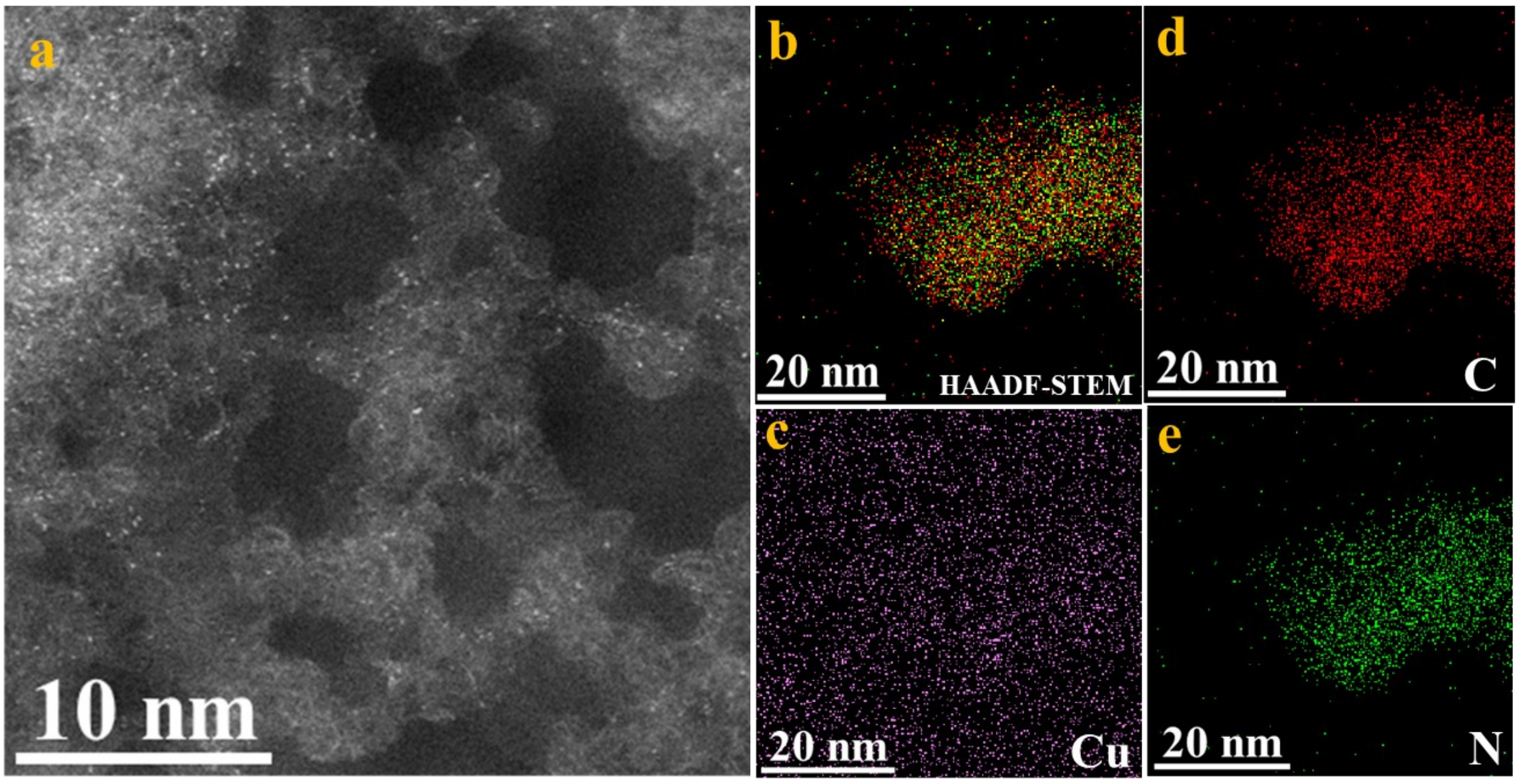

Fig S3. (a) HAADF-STEM of Cu-CN and (b-e) EDX elemental mapping of Cu, C, and N.

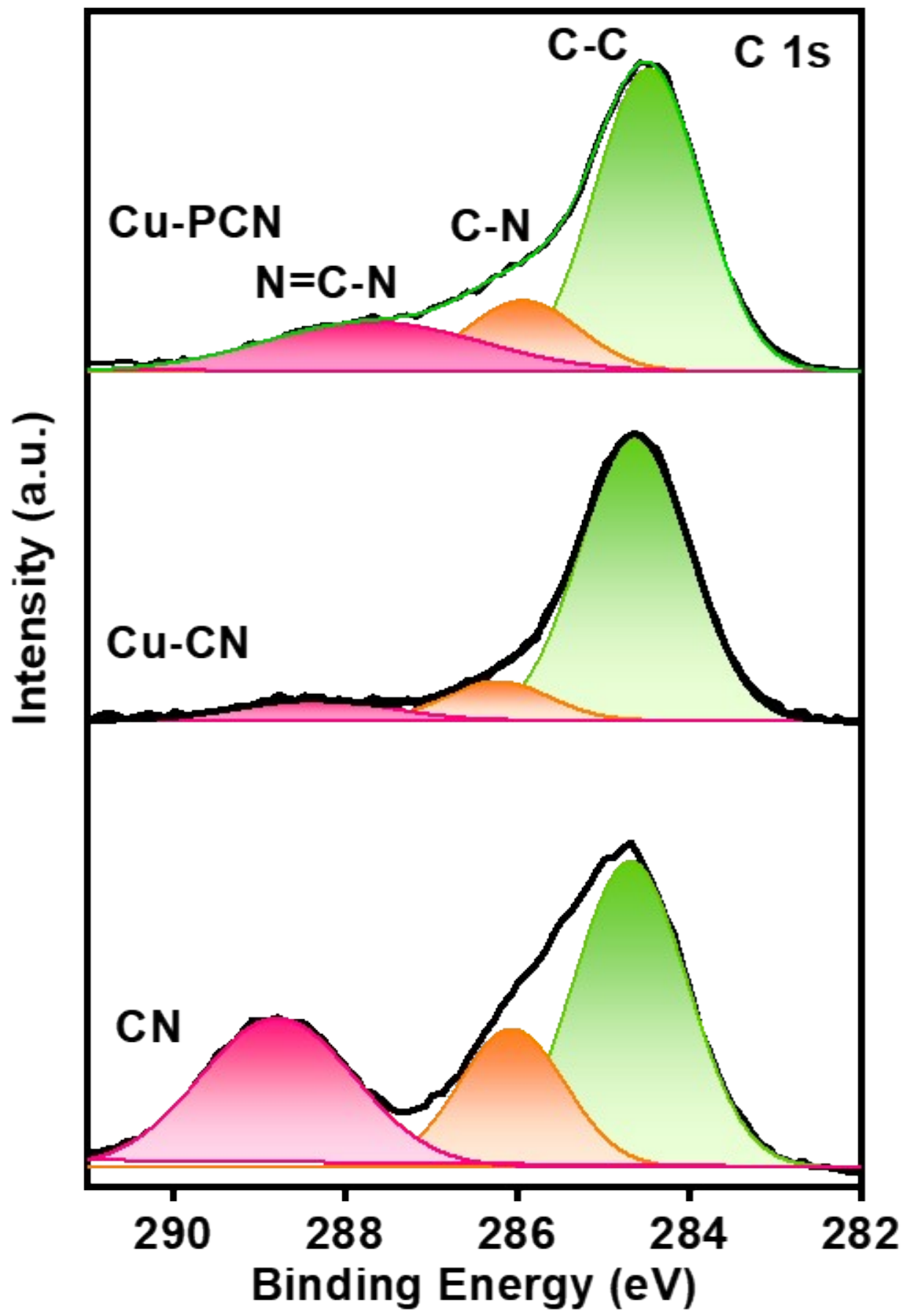

Fig S4. High-resolution C 1s XPS spectra of CN, Cu-CN, and Cu-PCN.

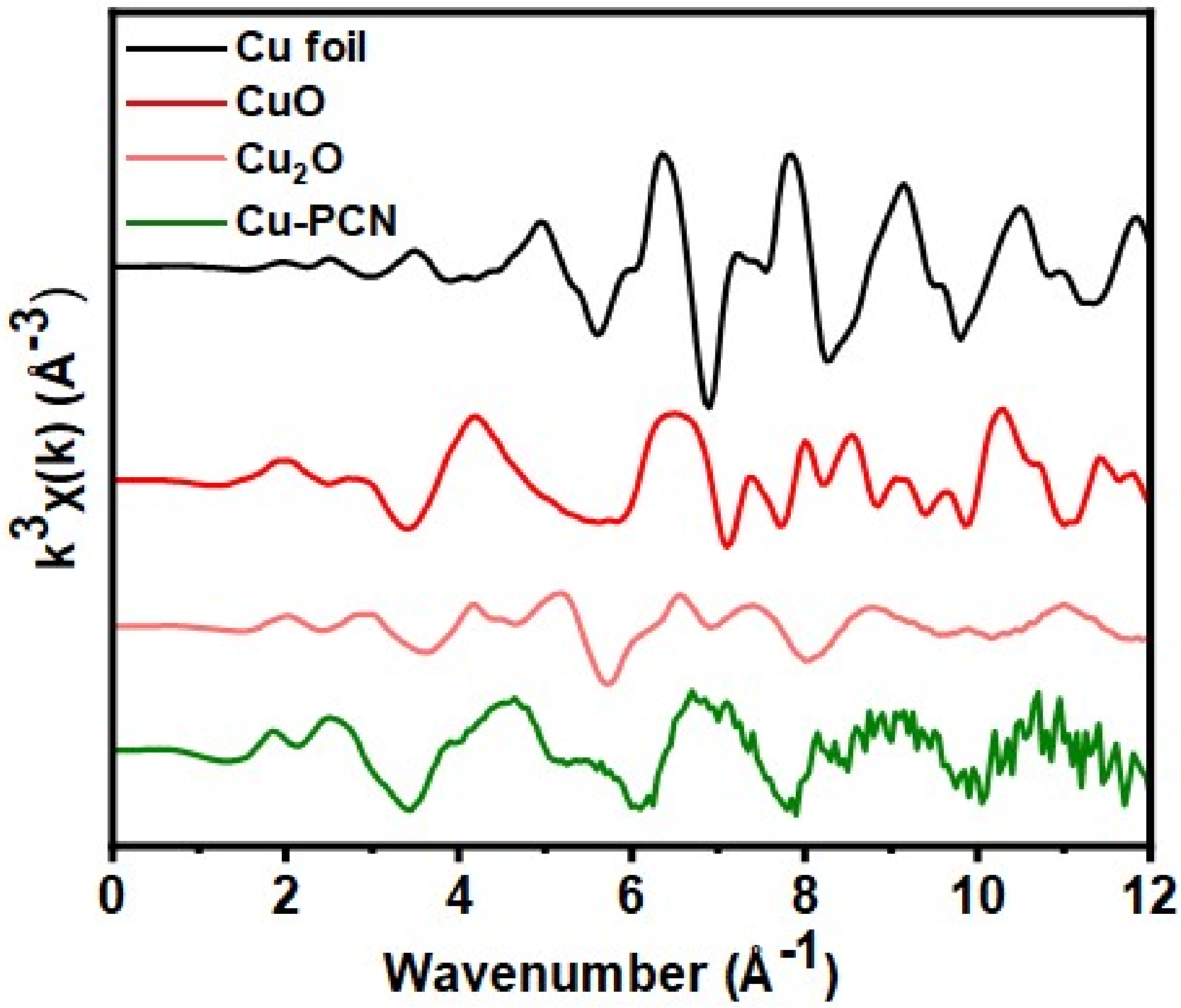

Fig S5. *k*-space EXAFS spectra of Cu-PCN and reference samples (Cu foil, Cu_2O, and CuO)

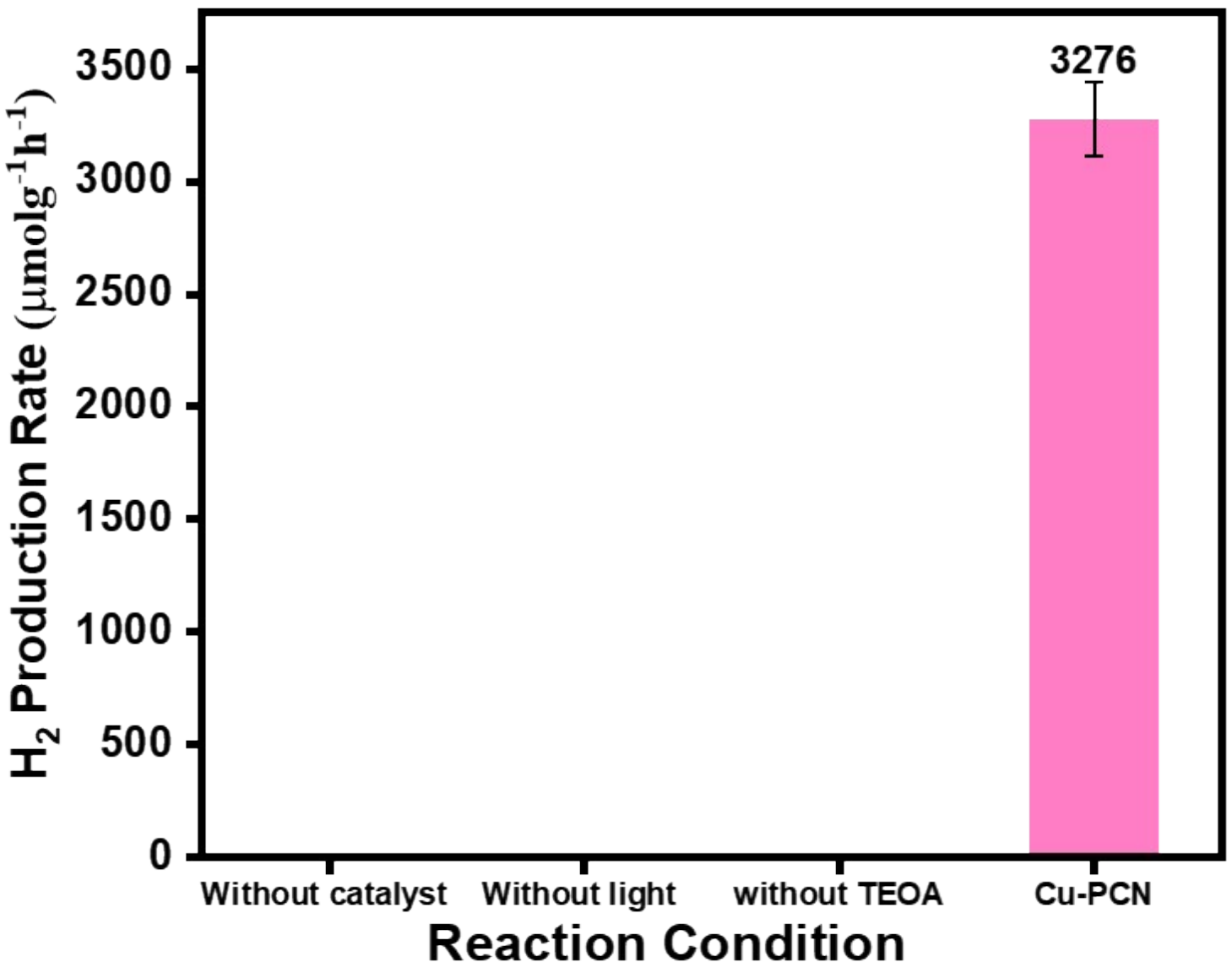

Fig S6. H_2 production with different reaction conditions

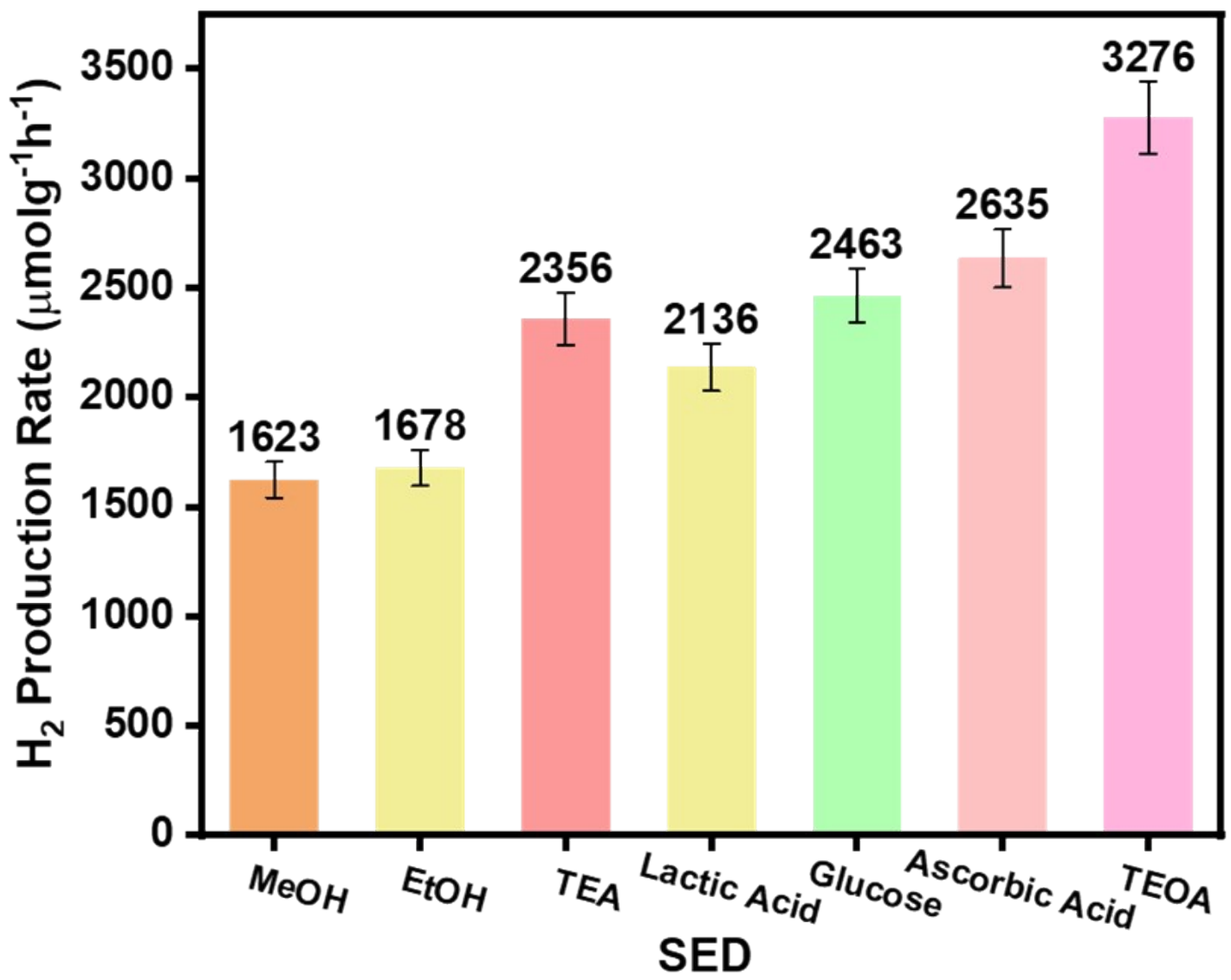

Fig S7. H_2 production with different sacrificial electron donors (SEDs).

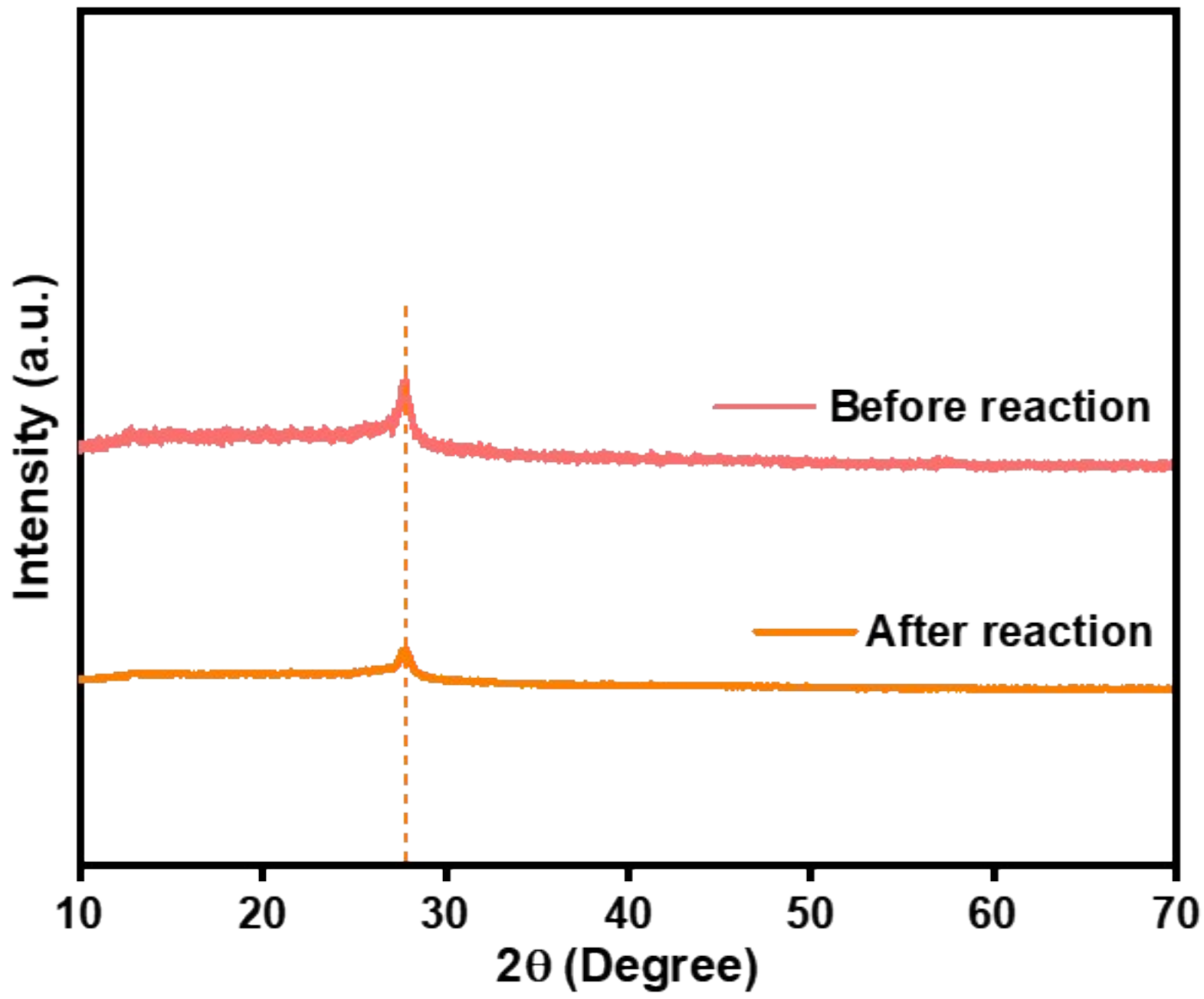

Fig S8. XRD of Cu-PCN before and after reaction.

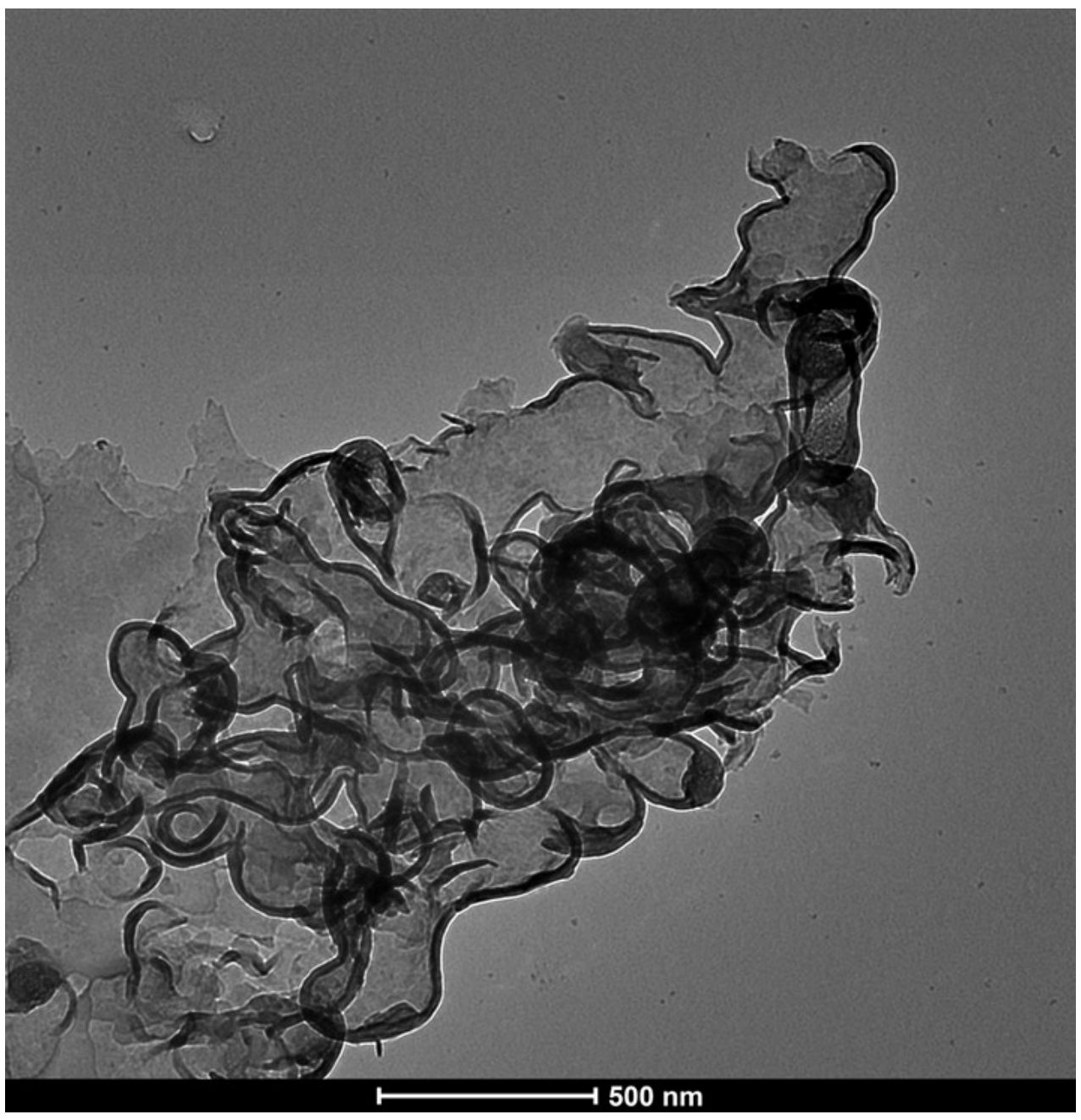

Fig S9. TEM image of Cu-PCN after reaction.

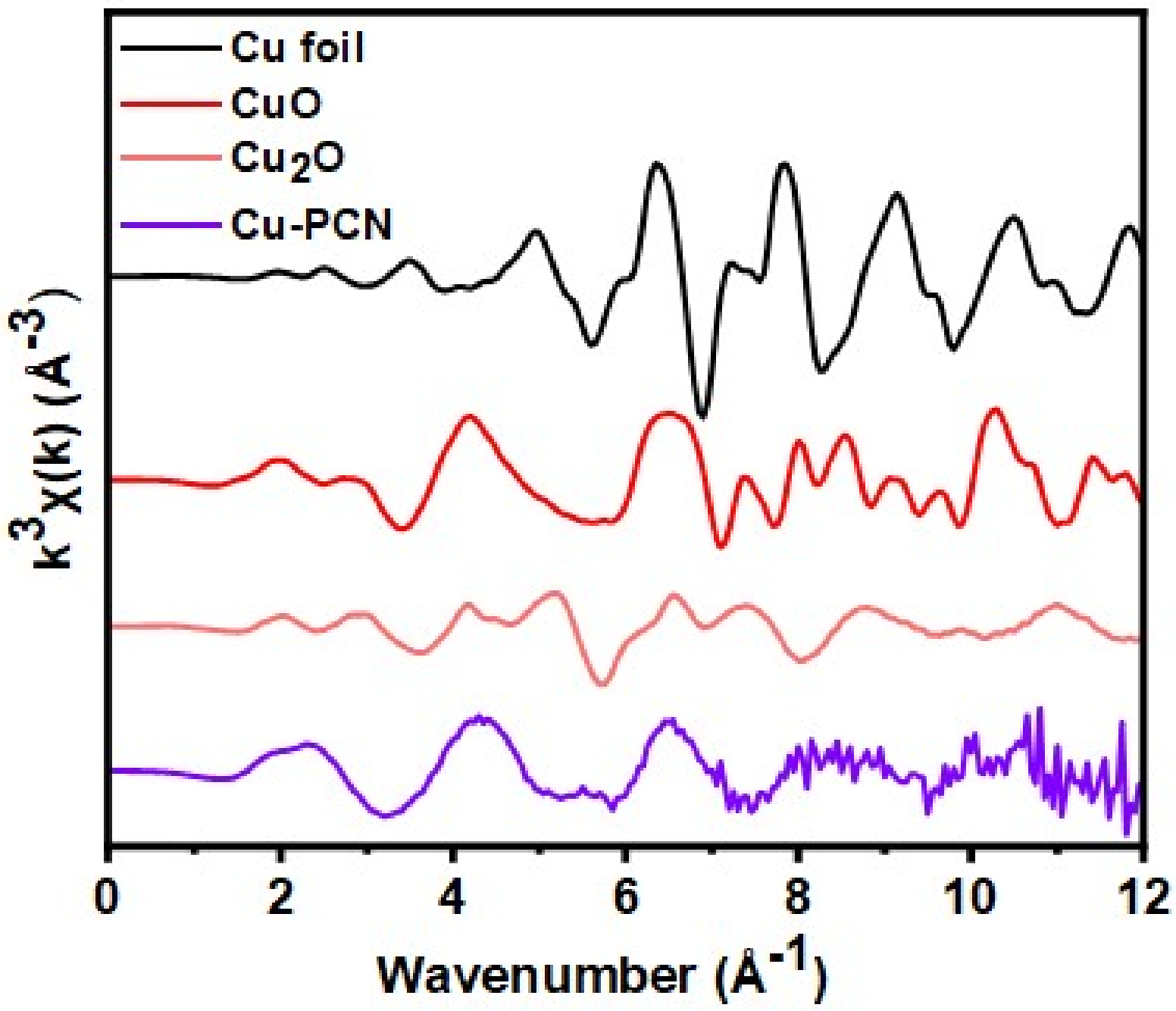

Fig S10. *k*-space EXAFS spectra of Cu-PCN after reaction and reference samples (Cu foil, Cu_2O, and CuO)

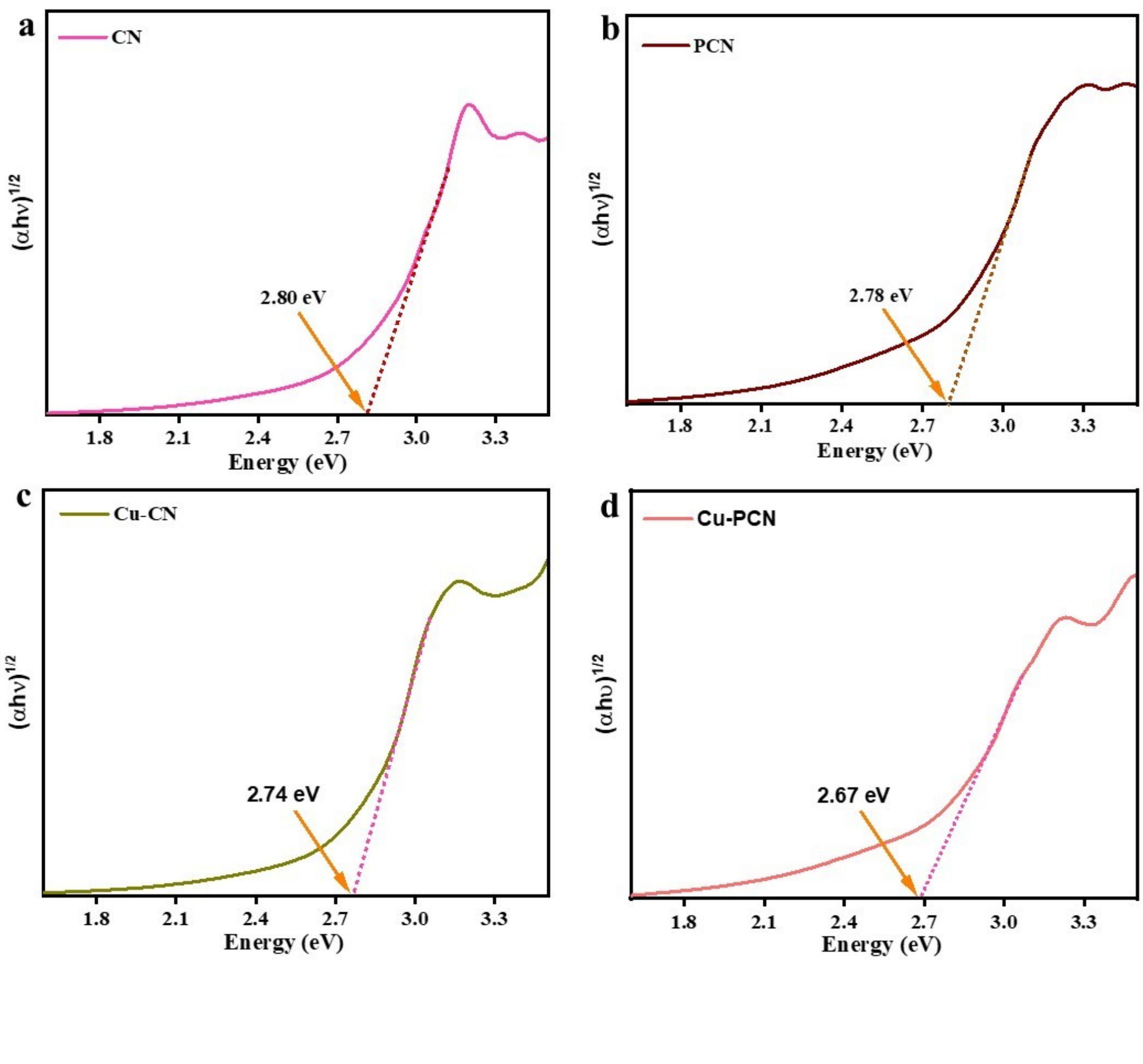

Fig S11. Tauc plot of (a) CN, (b) PCN, (c) Cu-CN and (d) Cu-PCN.

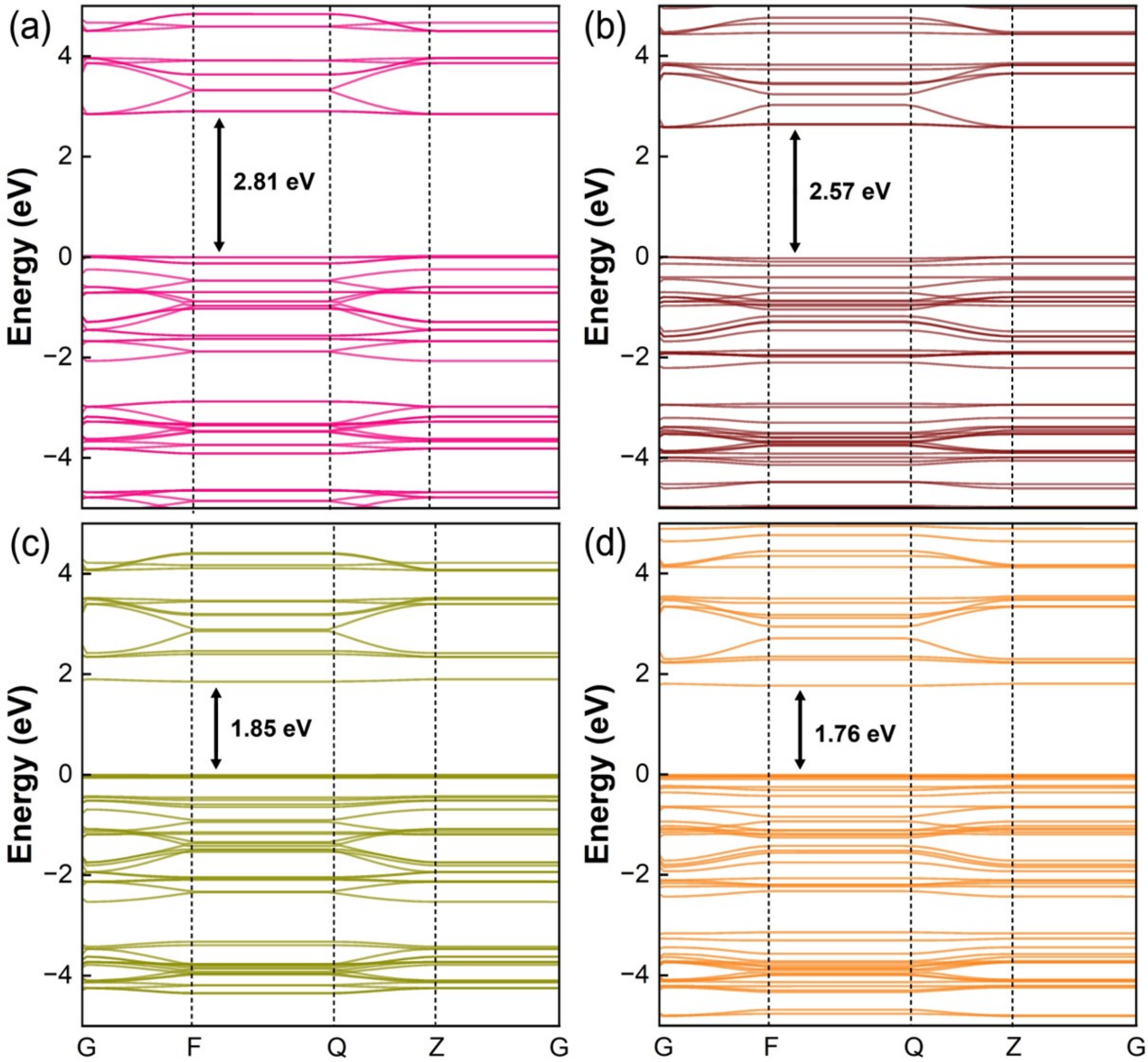

Fig S12. Calculated electronic band structures of (a) CN, (b) PCN, (c) Cu-CN, and (d) Cu-PCN

2.2. Supporting Tables

Table S1. The mass percentage from ICP-MS analysis for different samples

**Catalyst**	**Cu (wt%)**
Cu-CN	0.49
Cu-PCN	0.47

Table S2. The mass percentage from CHNS elemental analysis for different samples

**Catalyst**	**C(wt%)**	**N(wt%)**	**H(wt%)**	**C/N ratio**
CN	36.42	58.86	2.08	0.61
PCN	34.23	58.54	1.86	0.59
Cu-CN	36.33	59.12	2.17	0.61
Cu-PCN	34.16	58.32	2.12	0.58

Table S3. The ratio of peak areas for CN, Cu-CN and Cu-PCN based on XPS N-1s results.

**Catalyst**	**C=N-C**	**N-C_3**	**N-H**
CN	48.53	28.23	23.24
Cu-CN	51.58	29.19	19.22
Cu-PCN	55.38	28.27	16.34

Table S4. Comparison of g-C_3N_4-based photocatalysts for water splitting

**Catalyst**	**Light source**	**SED**	**AEQ (%)**	**H_2 evolution rate ($\mu molh^{-1}g^{-1}$)**	**Ref.**
**Cu-PCN**	**420 W Xe lamp**	**TEOA**	**31**	**3276**	**This work**
Pd/g- C_3N_4	300 W Xe lamp	TEOA	4	334.4	[8]
SA-Cu-CN-620	300 W Xe lamp	TEOA	66.38	605.14	[9]

RuCoP/g-C_3N_4	300 W Xe lamp	TEOA	3.49	1172.5	[10]
0.1Co_1Ag_1-PCN	300W Xe lamp	TEOA	1.49	1190	[11]
Cu_{SA}-g-C3N4	300W Xe lamp	TEOA	19.3	2142.4	[12]
hCNNS	300 W Xe lamp	TEOA	1.47	2380	[13]
Co@g-C_3N_4	300W Xe lamp	TEOA	5.04	2480	[14]
NCN-2AP-10	300 W Xe-lamp	TEOA	9.79	2550	[15]
PtSA-CN-620	300 W Xe lamp	TEOA	0.544	3020	[16]
HTCCN	300 W Xe lamp	TEOA		2017	[17]
SA-Cu-TCN	300 W Xe lamp	CH_3OH	9.2	212	[18]
Pt SACs g-C_3N_4	300 W Xe lamp	TEOA		1660	[19]
Fe@g-C3N4	300 W Xe lamp	TEOA	6.89	3390	[20]
BCN/PCN	300 W Xe lamp	TEOA		655	[21]

TEOA*: Triethanolamine. CH_3OH*: Methanol

Table S5. Lifetime parameters of CN, PCN, Cu-CN and Cu-PCN from the TRPL results.

**Sample**	**A_1 (%)**	**τ_1 (ns)**	**A_2 (%)**	**τ_2 (ns)**	**A_3 (%)**	**τ_3 (ns)**	**τ (ns)**
CN	10.4	5.65	9.8	0.82	9.8	1.62	4.41
PCN	10.9	6.82	9.9	0.92	9.2	1.90	5.43
Cu-CN	11.0	7.10	10.0	1.05	9.0	2.12	5.62

Cu-PCN	11.5	8.16	10.1	1.25	8.3	2.45	6.56

The average emission lifetime (τ) could be estimated through fitting the triexponential functions as followed.

$$\tau = \frac{A_1\tau_1^2 + A_2\tau_2^2 + A_3\tau_3^2}{A_1\tau_1 + A_2\tau_2 + A_3\tau_3}$$

References

1. G. Kresse and J. Furthmüller, Efficiency of ab-initio total energy calculations for metals and semiconductors using a plane-wave basis set, *Comput. Mater. Sci.,* 1996, ***6***, 15–50.
2. G. Kresse and J. Furthmüller, Efficient Iterative Schemes for Ab Initio Total-Energy Calculations Using a Plane-Wave Basis Set, *Phys. Rev. B*, 1996, **54**, 11169–11186.
3. P. E. Blöchl, Projector augmented-wave method, *Phys. Rev. B,* 1994, ***50***, 17953–17979..
4. J. P. Perdew, K. Burke and M. Ernzerhof, Generalized Gradient Approximation Made Simple, *Phys. Rev. Lett.*, 1996, **77**, 3865–3868.
5. H. J. Monkhorst and J. D. Pack, Special Points for Brillouin-Zone Integrations, *Phys. Rev. B*, 1976, **13**, 5188-5192.
6. S. Grimme, J. Antony, S. Ehrlich and H. Krieg, A Consistent and Accurate *ab initio* Parametrization of Density Functional Dispersion Correction (DFT-D) for the 94 Elements H–Pu, *J. Chem. Phys.,* 2010, **132**, 154104.
7. T. He, G. Gao, L. Kou, G. Will and A. Du, Endohedral metallofullerenes (M@C60) as efficient catalysts for highly active hydrogen evolution reaction, *J. Catal.,* 2017, **354**, 231–235.
8. S. Cao, H. Li, T. Tong, H.-C. Chen, A. Yu, J. Yu and H. M. Chen, Single-Atom Engineering of Directional Charge Transfer Channels and Active Sites for Photocatalytic Hydrogen Evolution, *Adv. Funct. Mater.,* 2018, **28**, 1802169.
9. J. Shen, C. Luo, S. Qiao, Y. Chen, Y. Tang, J. Xu, K. Fu, D. Yuan, H. Tang, H. Zhang and C. Liu, Single-Atom Cu Channel and N-Vacancy Engineering Enables Efficient Charge Separation and Transfer between C_3N_4 Interlayers for Boosting Photocatalytic Hydrogen Production, *ACS Catal.,* 2023, **13**, 6280–6288.
10. S. Meng, P. An, L. Chen, S. Sun, Z. Xie, M. Chen and D. Jiang, Integrating Ru-modulated CoP nanosheets binary co-catalyst with 2D g-C_3N_4 nanosheets for enhanced photocatalytic hydrogen evolution activity, *J. Colloid Interface Sci.,* 2021, ***585*,** 108–117.

11. Y. Liu, Y. Sun, E. Zhao, W. Yang, J. Lin, Q. Zhong, H. Qi, A. Deng, S. Yang, H. Zhang, H. He, S. Liu, Z. Chen and S. Wang, Atomically Dispersed Silver-Cobalt Dual-Metal Sites Synergistically Promoting Photocatalytic Hydrogen Evolution, *Adv. Funct. Mater.,* 2023, **33**, 2301840.
12. T. Zhou, H. Wei, B. Xiao, T. Lv, L. Duan, Q. Lu, J. Zhang, Y. Zhang and Q. Liu, Anchored Cu Single Atoms on Porous g-C_3N_4 for Superior Photocatalytic H_2 Evolution from Water Splitting, *RSC Adv.,* 2023, **13**, 8915-8922.
13. T. Li, S. Wang, L. Li, H. Zhu, Y. Yang and G. Liu, Constructing porous carbon nitride nanosheets for efficient visible-light-responsive photocatalytic hydrogen evolution , *J. Colloid Interface Sci.,* 2022, ***628*****,** 214-221.
14. W. Li, W. Li, Z. Guo, Y. Song, S. Tang, Y. Ma, X. Xing and Q. Wang, Synthesis of Atomically Thin g-C3N4 Nanosheets via Supercritical CO_2 Doping with Single-Atom Cobalt for Photocatalytic Hydrogen Evolution, *ACS Appl. Mater. Interfaces*, 2021, **13**, 52560-52570.
15. B. Ye, H. Tang, Q. Liu, W. Wang, L. Wang and J. Hu, I Extended π-conjugated system in carbon nitride by incorporating pyridine rings and N vacancies for photocatalytic H_2 evolution and H2O2 production, *Carbon,* 2023, **204**, 465-474.
16. Y. Zhang, C. Zhang, Y. Feng, T. Zhang, Q. Chen, Q. Chi, L. Liu, G. Li, Y. Cui, X. Wang, Z. Dang and Q. Lei, Excellent Energy Storage Performance and Thermal Property of Polymer-Based Composite Induced by Multifunctional One-Dimensional Nanofibers Oriented In-Plane Direction, *Nano Energy,* 2019, ***56***, 138-150.
17. Z. Zhao, Z. Shu, J. Zhou, T. Li, K. Wang and J. Song, Single LiCl-driven synthesis of heptazine-/triazine-based crystalline carbon nitride homojunction for enhanced solar hydrogen evolution, *J. Alloys Compd.*, 2023, **938**, 168484.
18. X. Xiao, Y. Gao, L. Zhang, J. Zhang, Q. Zhang, Q. Li, H. Bao, J. Zhou, S. Miao, N. Chen, J. Wang, B. Jiang, C. Tian and H. Fu, A Promoted Charge Separation/Transfer System from Cu Single Atoms and C_3N_4 Layers for Efficient Photocatalysis, *Adv. Mater.*, 2020, **32**, 2003082.
19. N. Lazaar, S. Wu, S. Qin, A. Hamrouni, B. Bikash Sarma, D. E. Doronkin, N. Denisov, H. Lachheb and P. Schmuki, Single-Atom Catalysts on C_3N_4: Minimizing Single Atom Pt Loading for Maximized Photocatalytic Hydrogen Production Efficiency, *Angew. Chem., Int. Ed.*, 2025, **64**, e202416453.
20. W. Zhang, Q. Peng, L. Shi, Q. Yao, X. Wang, A. Yu, Z. Chen and Y. Fu, Merging Single-Atom-Dispersed Iron and Graphitic Carbon Nitride to a Joint Electronic System for High-Efficiency Photocatalytic Hydrogen Evolution, *Small,* 2019, **15,** 1905166.

21. D. Yuan, Z. Li, X. Chen, J. Ding, H. Wan and G. Guan, Homodispersed B-CN/P-CN S-scheme homojunction for enhanced visible-light-driven hydrogen evolution, *Green Energy Environ,* 2022, **7**, 1119-1127.
